# Integrative taxonomy reveals three novel pathogens belonging to *Coniella*, *Colletotrichum* and *Pestalotiopsis* on *Nekemias grossedentata* in China

**DOI:** 10.3897/mycokeys.140.200783

**Published:** 2026-09-11

**Authors:** Shi-Qiang Chen, Xin-Tong Zhou, Xiao Mou, Shen-Xian Zhou, Qian Deng, Jian-Xin Deng, Hong Luo, Qing-Hua Chen, Hai-Feng Liu, Chao-Yang Zhang

**Affiliations:** 1 Department of Plant Protection, College of Agriculture, Yangtze University, Jingzhou 434025, China Department of Plant Protection, College of Agriculture, Yangtze University Jingzhou China; 2 Forewarning and Management of Agricultural and Forestry Pests, Hubei Engineering Technology Center, Yangtze University, Jingzhou 434025, China Forewarning and Management of Agricultural and Forestry Pests, Hubei Engineering Technology Center, Yangtze University Jingzhou China; 3 Enshi Tujia and Miao Autonomous Prefecture Academy of Agricultural Sciences, Enshi Tujia and Miao Autonomous Prefecture, Enshi 445000, China State Key Laboratory of Mycology, Institute of Microbiology, Chinese Academy of Sciences Beijing China; 4 State Key Laboratory of Mycology, Institute of Microbiology, Chinese Academy of Sciences, Beijing 100101, China Enshi Tujia and Miao Autonomous Prefecture Academy of Agricultural Sciences Enshi China

**Keywords:** *Ampelopsis grossedentata*, morphology, *Nekemias grossedentata*, novel species, phylogeny, species delimitation

## Abstract

*Nekemias grossedentata* (syn. *Ampelopsis grossedentata*, vine tea) is an economically important medicinal plant in China, yet the diversity of foliar fungal pathogens remains poorly understood. In this study, three pathogenic fungi were isolated from symptomatic *N. grossedentata* leaves collected in Enshi Tujia and Miao Autonomous Prefecture, Hubei Province, China. Their taxonomic status was determined using an integrative approach combining morphological characterisation, multi-locus phylogenetic analysis, coalescent species delimitation and SNP-based PCA (SNPs extracted from concatenated multi-locus sequences). The pathogenicity was confirmed by fulfilling Koch’s postulates. Three novel species are formally described herein: *Coniella ampelopsidis*, *Colletotrichum enshiense* and *Pestalotiopsis ampelopsidis*. All analyses consistently resolved the three species as independent taxa with highly-supported evolutionary lineages, distinct genetic clustering and significant nucleotide divergence. This study also enriches the inventory of foliar fungal pathogens infecting *Nekemias grossedentata* and provides a basis for integrated disease management.

## Introduction

*Nekemias grossedentata* (syn. *Ampelopsis grossedentata*, vine tea) is an economically important edible and medicinal vine plant in the *Vitaceae* family (Wen et al. 2014; Li et al. 2021), with a natural distribution primarily across southern China (Wu et al. 2023), of which Enshi Prefecture is the core production area of vine tea. This plant is the leading agricultural product of Enshi and Enshi Vine Tea (leaf tea made from *N. grossedentata*) is one of the National Geographical Indication Products (Xi et al. 2025). Fungal diseases seriously impair plant quality and yield, inducing leaf spots, blight symptoms and even whole-plant mortality (Yuan et al. 2022; Liu et al. 2024). Four species have been documented: *Phomopsis viticola* (Yi et al. 2019), *Pestalotiopsis microspora* (Yuan et al. 2022), *Diaporthe eres* (Liu et al. 2024) and *Colletotrichum nymphaeae* (Shi et al. 2025). A recent study reported a novel *Coniella* species (*Coniella grossedentatae*) on *N. grossedentata* without pathogenicity assessment (Li et al. 2025).

The fungal taxonomy has evolved from relying solely on morphology to multi-locus phylogeny, greatly improving accuracy and species resolution. Early classification relied primarily on conidial phenotypic characters, but high morphological similarity amongst intergeneric species makes accurate identification based solely on phenotype challenging (Taylor et al. 2000). DNA sequence-based phylogeny has become the core approach to taxonomy, using standardised genus-specific multi-locus barcodes: internal transcribed spacer (ITS), large subunit ribosomal RNA gene (LSU), DNA-dependent RNA polymerase subunit II (*RPB2*), translation elongation factor 1-alpha (*TEF1*) for *Coniella* (Alvarez et al. 2016); ITS, chitin synthase 1 (*CHS*), actin (*ACT*), calmodulin (*CAL*) and beta-tubulin (*TUB2*) for *Colletotrichum* (Damm et al. 2012); and ITS, *TEF1* and *TUB2* for *Pestalotiopsis* (Maharachchikumbura et al. 2014; Zhang et al. 2026). However, the species boundary assignment according to concatenated tree topologies has inherent limitations and a non-negligible risk of false positives (Cai et al. 2011). Modern fungal taxonomy adopts an integrative multi-evidence framework combining morphological characterisation and core multi-locus phylogeny for basic taxonomic structure; phylogenetic networks and SNP-based PCA for fine-scale genetic differentiation; gene flow detection and cross-validation of GCPSR, DBSR and CBSR for robust species boundaries (Druzhinina et al. 2012; Maharachchikumbura et al. 2021; Pereira and Phillips 2024).

In 2025, a severe foliar disease on cultivated *N. grossedentata* occurred in plantations of Enshi Prefecture, Hubei Province. Fungal pathogenic species were isolated from diseased leaf symptoms, determined and described using the integrative multi-evidence framework.

## Materials and methods

### Sample collection and fungal isolation

**Figure 1.**
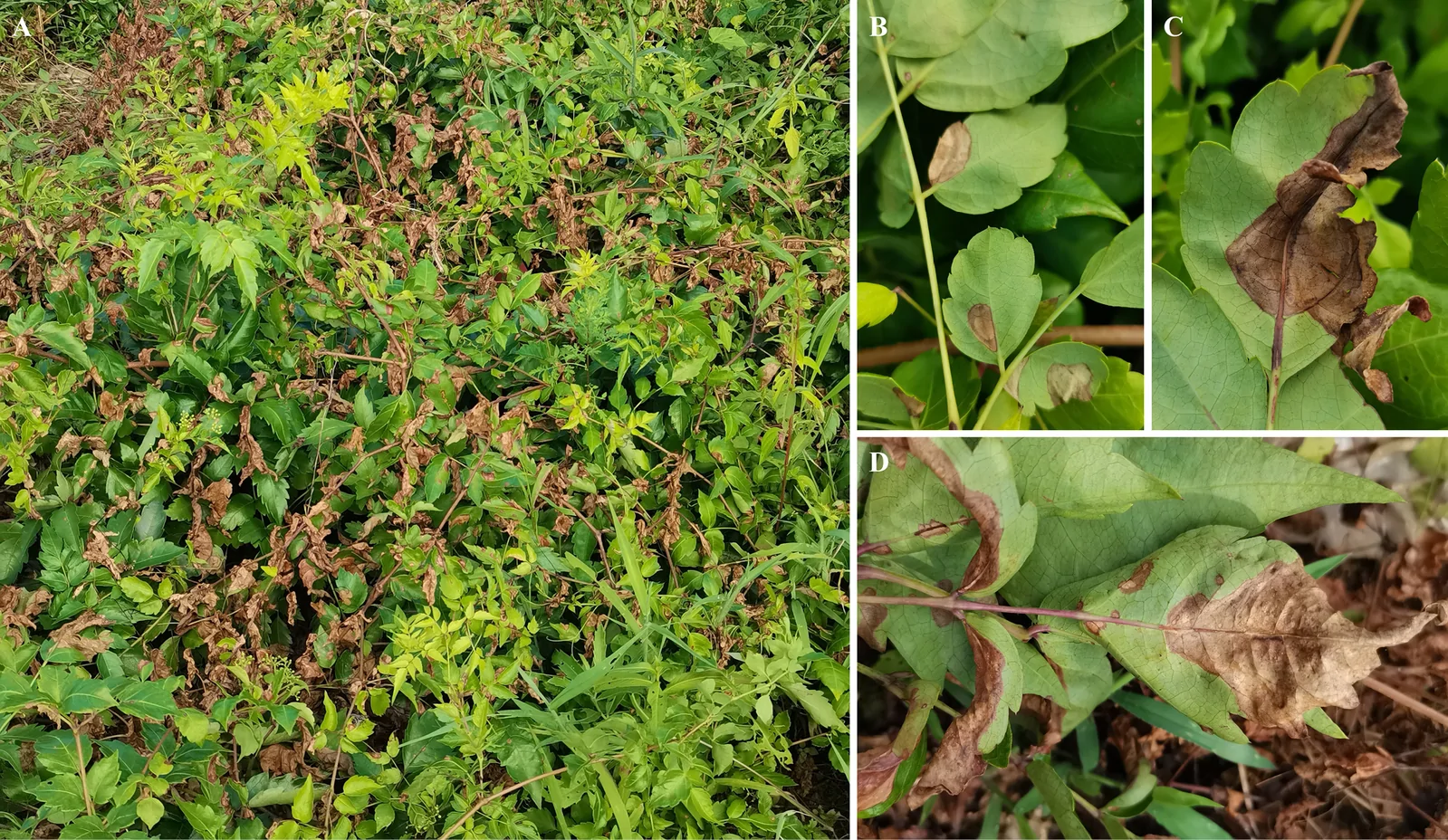
Natural foliar disease symptoms on *Nekemias grossedentata* in the field. **A**. General view of diseased plants in the plantation; **B–D**. Close-up views of different morphological types of necrotic lesions on infected leaves.

In 2025, multi-site sampling was conducted in *N. grossedentata* plantations in Enshi Prefecture, Hubei, China (30°19'46.41"N, 109°32'20.20"E, 575 m above sea level). A total of 83 samples with clear disease symptoms (Fig. 1) were obtained and transported to the laboratory for fungal isolation. Tissue segments (1 cm^2^) containing both necrotic and healthy tissue were excised from typical single lesions, surface-sterilised in 75% ethanol (1 min), 2% sodium hypochlorite (30 s), rinsed three times in sterile distilled water and cultured on to potato dextrose agar (PDA, BD Difco, Montreal, Canada). Single hyphal tip was transferred to fresh PDA plates and purified, from which 116 pure cultures were preserved in PDA slants (4 °C) and glycerol stocks (-80 °C), deposited in the Yangtze University Fungal Herbarium, Jingzhou, Hubei, China.

### Morphological observations

Colony characteristics, including growth rate, colour and texture, were determined on PDA. Mycelial plugs (6 mm in diameter) were cut from the margins of active PDA colonies, inoculated on to new fresh PDA and grown in the dark at 25 °C. Colony features were recorded after 7 days.

To observe conidial morphology (conidiogenesis, colour, shape, size) and reproductive structures, different culture conditions were used. *Coniella* and *Pestalotiopsis* species were cultured on PDA at 25 °C under a 12 h light/12 h dark cycle for 14 days to induce sporodochial formation and conidial production (Maharachchikumbura et al. 2014; Mahadevakumar et al. 2022). Cross-sections of sporodochia were prepared using a cryostat (Epredia, Kalamazoo, MI, USA) to examine internal sporogenous structures. *Colletotrichum* species were cultured on oatmeal agar (OA; Difco, Montreal, Canada) under identical conditions for 14 days to observe sporogenous structures and conidia (Hassan et al. 2022; Norphanphoun et al. 2025).

Mounts were prepared in Lactophenol Picric Acid (LPA) or Lactophenol Cotton Blue (LPCB) solution. Micrographs were captured using a Nikon ECLIPSE Ni-U microscope (Nikon Corporation, Tokyo, Japan). Morphological measurements were based on 50 randomly selected conidia per strain.

### DNA extraction and PCR amplification

Total genomic DNA was extracted from fresh mycelia harvested from PDA using the cetyltrimethylammonium bromide (CTAB) method (Porebski et al. 1997). Polymerase chain reaction (PCR) amplifications were performed in 25 μl reaction volumes containing 21 μl of 1.1× Taq PCR Star Mix (TSINGKE, Beijing, China), 2 μl of template DNA and 1 μl each of forward and reverse primers. PCR thermal cycling was carried out on a Bio-Rad T100 thermal cycler (Bio-Rad, California, USA). The primers and detailed reaction conditions for amplification of the ITS, LSU, *RPB2*, *TEF1*, *CHS*, *ACT*, *CAL* and *TUB2* loci are listed in Table 1. The resulting PCR products were bidirectionally sequenced using the Sanger method by Tsingke Biotechnology.

**Table 1.** PCR primer pairs used in the study and their related references.

**Gene region**	**Primer pair**	**Sequence (5'-3')**	**PCR: Thermal Cycles**	**Reference**
ITS	ITS5	GGAAGTAAAAGTCGTAACAAGG	(95 °C: 30s, 51 °C: 30s, 72 °C: 1 min) × 35 cycles	White et al. (1990)
	ITS4	TCCTCCGCTTATTGATATGC		
*RPB2*	RPB2-5F	GAYGAYMGWGATCAYTTYGG	(95 °C: 15s, 54 °C: 20s, 72 °C: 1 min) × 35 cycles	Sung et al. (2007)
	RPB2-7CR	CCCATRGCTTGTYYRCCCAT		
*TEF1*	EF1-728F	CATCGAGAAGTTCGAGAAGG	(95 °C: 15s, 54 °C: 20s, 72 °C: 1 min) × 35 cycles	Carbone and Kohn (1999)
	EF1-986R	TACTTGAAGGAACCCTTACC		
*β-TUB*	Bt2a	GGTAACCAAATCGGTGCTGCTTTC	(95 °C: 15s, 54 °C: 20s, 72 °C: 1 min) × 35 cycles	Glass and Donaldson (1995)
	Bt2b	ACCCTCAGTGTAGTGACCCTTGGC		
LSU	LR0R	ACCCGCTGAACTTAAGC	(95 °C: 30s, 55 °C: 30s, 72 °C: 1 min) × 35 cycles	Vilgalys and Hester 1990; Rehner and Samuels (1994)
	LR5	TCCTGAGGGAAACTTCG		
*CAL*	CL1C	GAATTCAAGGAGGCCTTCTC	(95 °C: 15s, 54 °C: 20s, 72 °C: 1 min) × 35 cycles	Carbone and Kohn (1999)
	CL2C	CTTCTGCATCATGAGCTGGAC		
*CHS*	79F	TGGGGCAAGGATGCTTGGAAGAAG	(95 °C: 30s, 58 °C: 30s, 72 °C: 1 min) × 35 cycles	Carbone and Kohn (1999)
	354R	TGGAAGAACCATCTGTGAGAGTTG		
*ACT*	512F	ATGTGCAAGGCCGGTTTCGC	(95 °C: 45s, 55 °C: 45s, 72 °C: 1 min) × 35 cycles	Carbone and Kohn (1999)
	783R	TACGAGTCCTTCTGGCCCAT		

## Integrative taxonomy species delimitation

### Phylogenetic analyses

Sequences were analysed using Chromas 2.6.6 and contig assembly was performed using Phydit 3.2 (Chun 1995). Reference sequences were retrieved from the NCBI GenBank database, based on BLAST results (Suppl. material 1). Separate multi-locus datasets were constructed for the three fungal genera (*Coniella*: ITS, LSU, *RPB2*, *TEF1*; *Colletotrichum*: ITS, *ACT*, *CAL*, *CHS*, *TUB2*; *Pestalotiopsis*: ITS, *TEF1*, *TUB2*) and MEGA 11.0.11 and OFPT 1.9.0 (Tamura et al. 2021; Zeng et al. 2023) were used for the concatenated sequence alignments. Phylogenetic constructions were performed using Maximum Likelihood (ML) and Bayesian Inference (BI) methods. Phylogenetic networks were constructed using SplitsTree 4.19.2 (Quaedvlieg et al. 2014).

Nucleotide substitution models for both ML and BI analyses were selected by MrModelTest v.2.3, based on the Akaike Information Criterion (AIC) (Rannala and Yang 1996). ML bootstrap support (BS) was estimated with 1000 replicates. For BI, two independent Markov Chain Monte Carlo (MCMC) chains were run for 1,000,000 generations, sampling every 100 generations. The first 25% of trees were discarded as burn-in and posterior probabilities (PP) were calculated from the 50% majority-rule consensus tree. Trees were visualised and edited in FigTree 1.3.1 (Rambaut 2009), with branches annotated for BS ≥ 70% and PP ≥ 0.8. Phylogenetic networks were constructed from the same alignments using LogDet transformation and split decomposition (Huson and Bryant 2006).

### Species delimitation

Species boundaries were inferred using an integrative framework incorporating Genealogical Concordance Phylogenetic Species Recognition (GCPSR), Distance-Based Species Recognition (DBSR) and Concordance-Based Species Recognition (CBSR) (Taylor et al. 2000; Puillandre et al. 2012, 2021; Vences et al. 2021). The genetic datasets used for species delimitation analyses were identical to those used for phylogenetic reconstructions.

For GCPSR, topological congruence across individual single-gene genealogies was evaluated to verify the stability and support of candidate monophyletic lineages. Recombination events were evaluated for each locus using the Pairwise Homoplasy Index (PHI) test in SplitsTree 4.19.2 (Bruen et al. 2006; Huson and Bryant 2006), with P < 0.05 denoting a significant recombination signal. For CBSR, Poisson Tree Processes (PTP) and Bayesian PTP (bPTP) analyses were executed via iTaxoTools (PTP advanced 0.1) using the ML tree topology as the input tree, running for 1,000,000 MCMC generations with a 25% burn-in. For DBSR, single-locus alignments were analysed independently using Automatic Barcode Gap Discovery (ABGD) and Assemble Species by Automatic Partitioning (ASAP) implemented in iTaxoTools GUI (iTaxoTools ABGD py 0.1 and iTaxoTools ASAPy 1.0) under the K2-P model (Kimura parameter = 2.0) and otherwise default parameter settings (Vences et al. 2021; Zhang et al. 2026).

A hierarchical decision framework was applied to establish species boundaries, GCPSR: Putative clades were required to exhibit genealogical concordance across non-recombinational single-gene genealogies (verified by the PHI P < 0.05) (Taylor et al. 2000; Bruen et al. 2006); Multi-algorithm Consensus Criterion: a species boundary was formally accepted if corroborated by at least three independent delimitation methods across distance-based and coalescent-based models (PTP, bPTP, ASAP, ABGD) under a majority-consensus strategy to minimise single-method bias (Carstens et al. 2013; Maharachchikumbura et al. 2021); Distance-Score Priority Rule: for distance-based species recognition methods, ASAP partitions with lower metrics/confidence scores were prioritised over ABGD when barcode gap boundaries were ambiguous (Puillandre et al. 2012, 2021). Ultimately, a lineage was validated as a robust species if the integrated species delimitation consensus aligned with the multi-locus phylogenetic tree topologies (ML/BI) (Dissanayake et al. 2024; Hyde et al. 2024).

### SNP-PCA (Single Nucleotide Polymorphism-based Principal Component Analysis)

SNP-PCA was performed to assess genetic differentiation amongst the species. SNPs were extracted from the concatenated multi-locus sequence alignments using SNP-sites, a programme designed for rapid identification of SNPs from multi-FASTA alignments (Page et al. 2016). The resulting SNP matrix was imported into R and PCA was conducted using the prcomp function implemented in the base *stats* package (R Core Team 2024). The first two principal components were visualised as scatter plots using the ggplot2 package (Wickham 2016).

### Pathogenicity tests

Pathogenicity assays were performed on leaves of *N. grossedentata*. Plants were acclimatised in a greenhouse (25 °C, 12 h photoperiod) for 2 weeks. After surface-disinfecting the leaf surfaces with 75% ethanol, living leaves were inoculated with 7-day-old PDA mycelial plugs (6 mm), sterile PDA plugs as controls. Three biological replicates (3 leaves each) per strain were tested, which was conducted in three independents. Plants were kept in high humidity (> 85%) and symptom appearance recorded daily. Pathogenicity differences were evaluated at 7 dpi by measuring lesion size (n = 27). Koch’s postulates were confirmed by re-isolation and the ITS sequences. Data are shown as mean ± SD. Normality and homogeneity of variances were confirmed using Shapiro-Wilk and Levene’s tests (Shapiro and Wilk 1965). Lesion area differences amongst strains were compared using one-way ANOVA and Tukey’s HSD post hoc test (Levene 1960). All tests were two-tailed at α = 0.05 and analyses were conducted in IBM SPSS Statistics 24.0.0 (George and Mallery 2019).

## Results

### Phylogenetic Analyses

**Figure 2.**
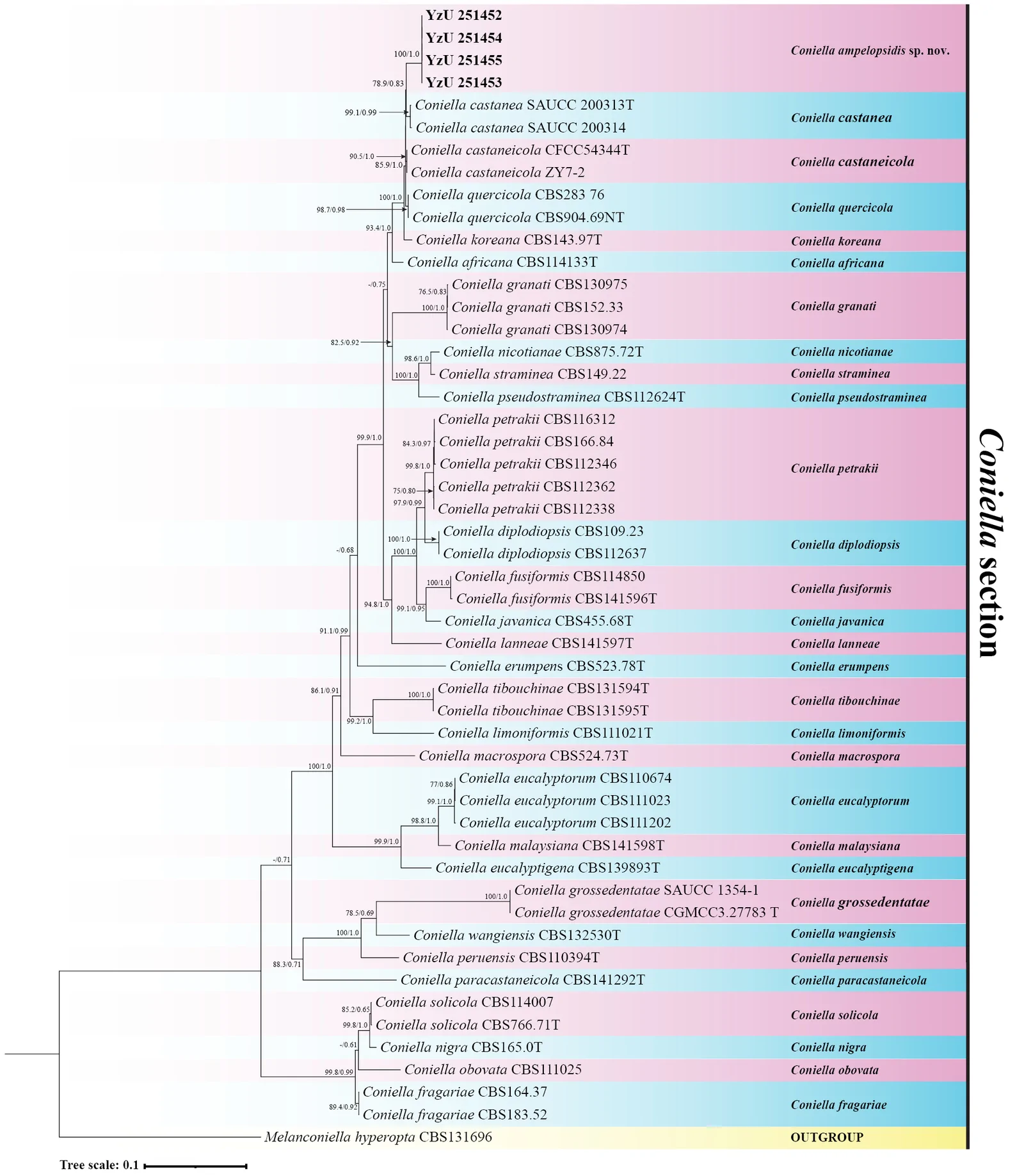
Phylogenetic tree, based on the combined gene sequences of ITS, LSU, *RPB2* and *TEF1* generated from *Coniella* sp. on *Nekemias grossedentata*. The Maximum Likelihood bootstrap values (BS > 70%) and Bayesian posterior probabilities (PP > 0.80) are provided for the nodes (PP/BS). The current strains are in bold.

**Figure 3.**
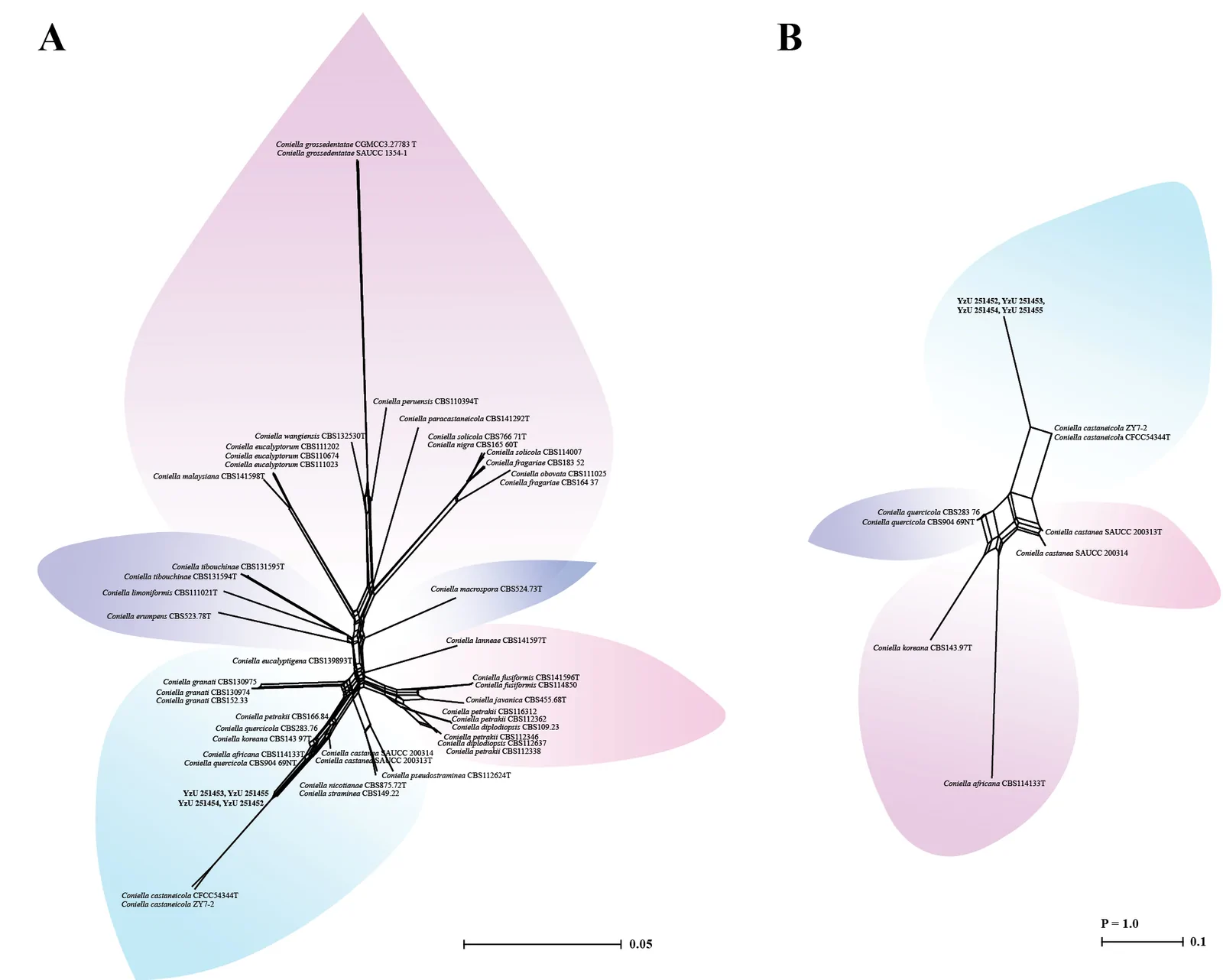
Neighbour-Net phylogenetic network of the genus *Coniella*, based on the concatenated ITS, LSU, *RPB2* and *TEF1*. The network was constructed using LogDet transformation and the NeighbourNet algorithm implemented in SplitsTree4. **A**. Overall network showing the phylogenetic position of the new species; **B**. Enlarged view of the clade containing the new species and its closely-related congeners. Sequences generated in this study are shown in bold.

***Coniella***: The dataset included 2793 aligned characters (ITS: 662, LSU: 894, *TEF1*: 362, *RPB2*: 721). Best-fit substitution models were used for different gene regions: SYM+I+G for ITS, GTR+I+G for LSU/*RPB2* and HKY+I+G for *TEF1* (Alvarez et al. 2016). The ML and BI topologies were congruent with each other and the new species delimitation was supported by phylogenetic networks (Figs 2, 3A, B). The ML phylogeny was used as the basal tree. Four strains (YzU 251452, YzU 251453, YzU 251454 and YzU 251455) formed a distinct monophyletic clade within *Coniella* as a sister group to *C. castanea* without internal reticulate branches, with highly supported BS/PP (100/1.0) values. Besides, a non-overlapping genetic boundary existed between these two clades further confirming the genetic independence of the present strains as a novel species.

**Figure 4.**
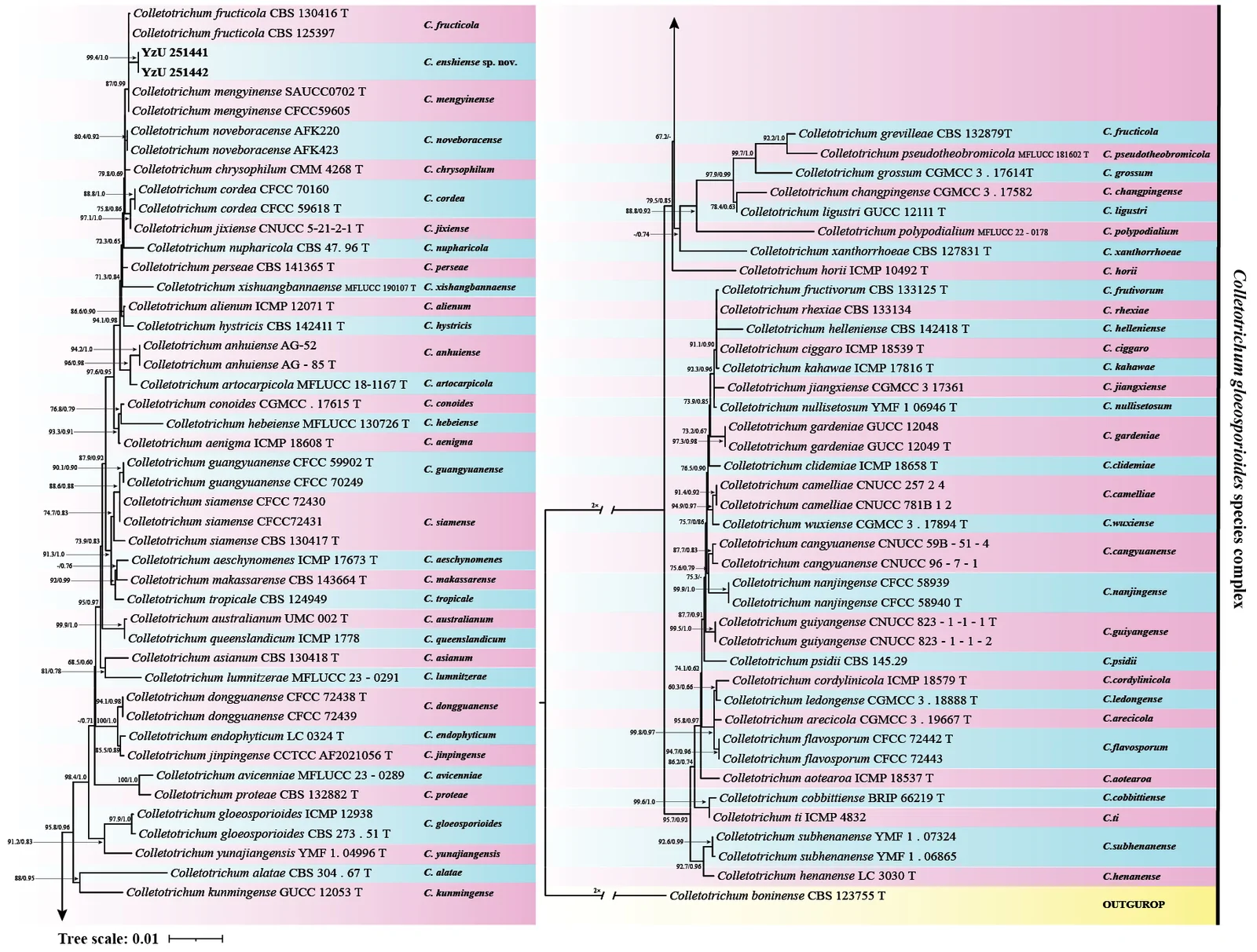
Phylogenetic tree, based on combined gene sequences of ITS, *ACT*, *CAL*, *CHS* and *TUB2* from the *Colletotrichum gloeosporioides* species complex. The Maximum Likelihood bootstrap values (BS > 70%) and Bayesian posterior probabilities (PP > 0.80) are provided for the nodes (PP/BS). The current strains are in bold.

**Figure 5.**
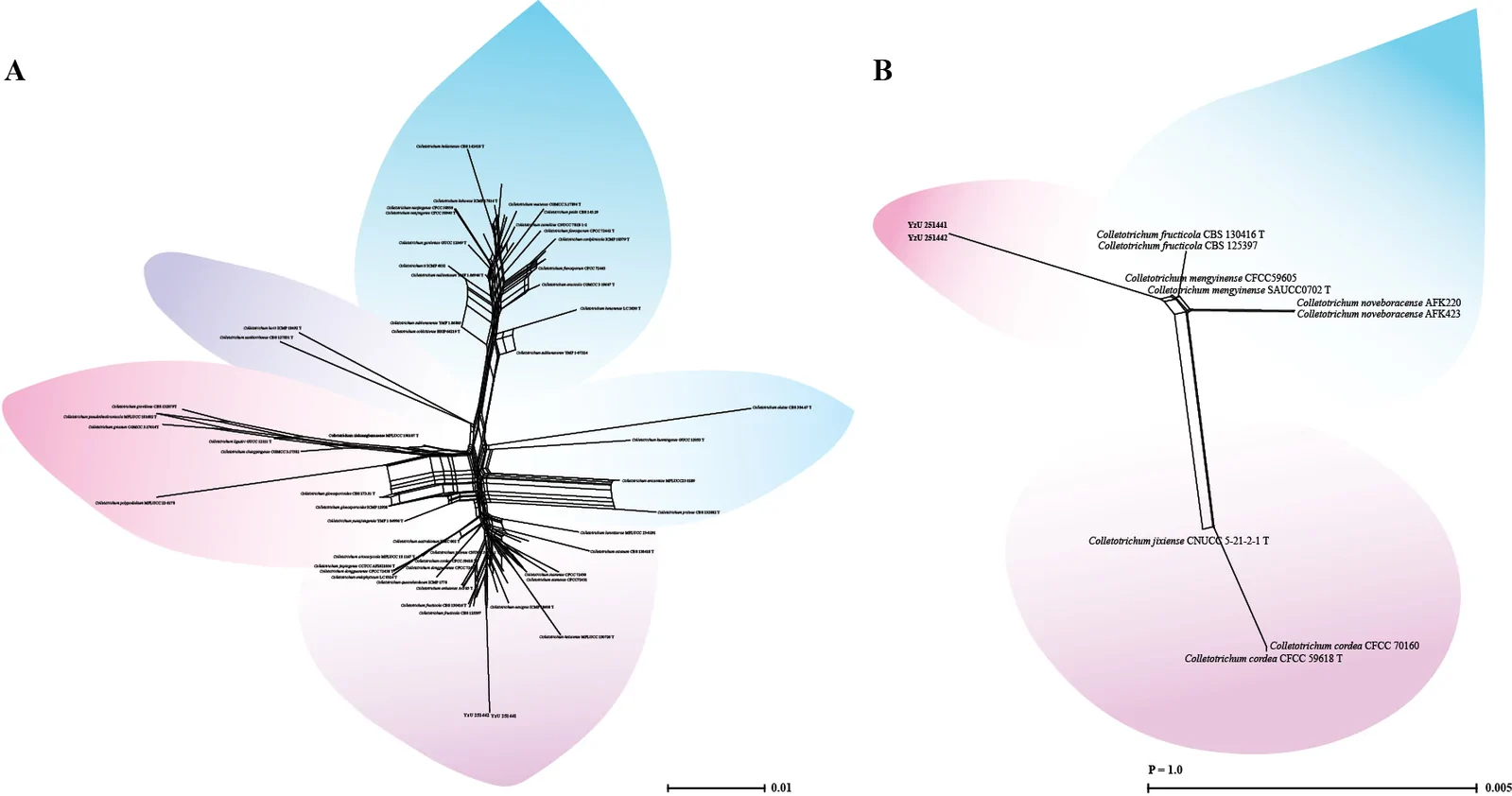
Neighbour-Net phylogenetic network of the genus *Colletotrichum gloeosporioides* species complex, based on the concatenated ITS, *ACT*, *CAL*, *CHS* and *TUB2*. The network was constructed using LogDet transformation and the NeighbourNet algorithm implemented in SplitsTree4. **A**. Overall network showing the phylogenetic position of the new species; **B**. Enlarged view of the clade containing the new species and its closely-related congeners. Sequences generated in this study are shown in bold.

***Colletotrichum***: Multi-locus phylogenetic trees (ITS, *CHS*, *ACT*, *CAL*, *TUB2*) were constructed to resolve the taxonomic status of *Colletotrichum*. The dataset included 2344 aligned characters (ITS: 662, *ACT*: 238, *CHS*: 229, *CAL*: 749, *TUB2*: 478). Best-fit substitution models: GTR+I+G for ITS, *ACT* and *CHS*, HKY+I+G for *CAL* and *TUB2* (Guarnaccia et al. 2017). Congruent ML/BI topologies supported by phylogenetic networks (Figs 4, 5A, B). The ML tree was used as reference. The tested strains (YzU 251441, YzU 251442) formed a distinct monophyletic clade (BS/PP = 100/1.0) within *Colletotrichum* as a sister to *Col. fructicola* and *Col. mengyinense*, no internal reticulation without overlapping genetic boundary. Phylogenetic results indicate these two isolates are conspecific and represent a novel distinct taxon.

**Figure 6.**
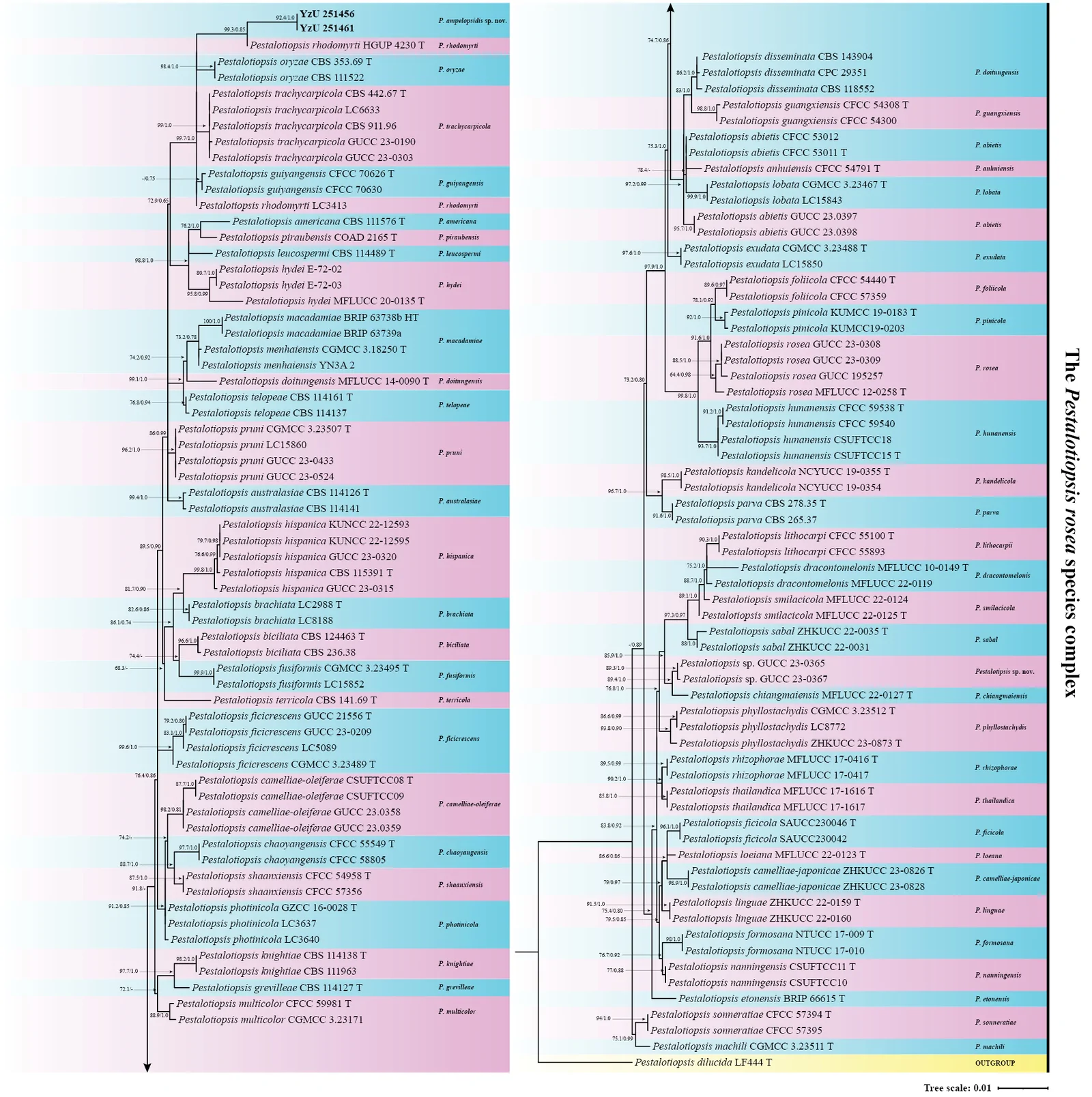
Phylogenetic tree, based on the combined gene sequences of ITS, *TEF1* and *TUB2* generated from *Pestalotiopsis* sp. on *Nekemias grossedentata*. The Maximum Likelihood bootstrap values (BS > 70%) and Bayesian posterior probabilities (PP > 0.80) are provided for the nodes (PP/BS). The current strains are in bold.

**Figure 7.**
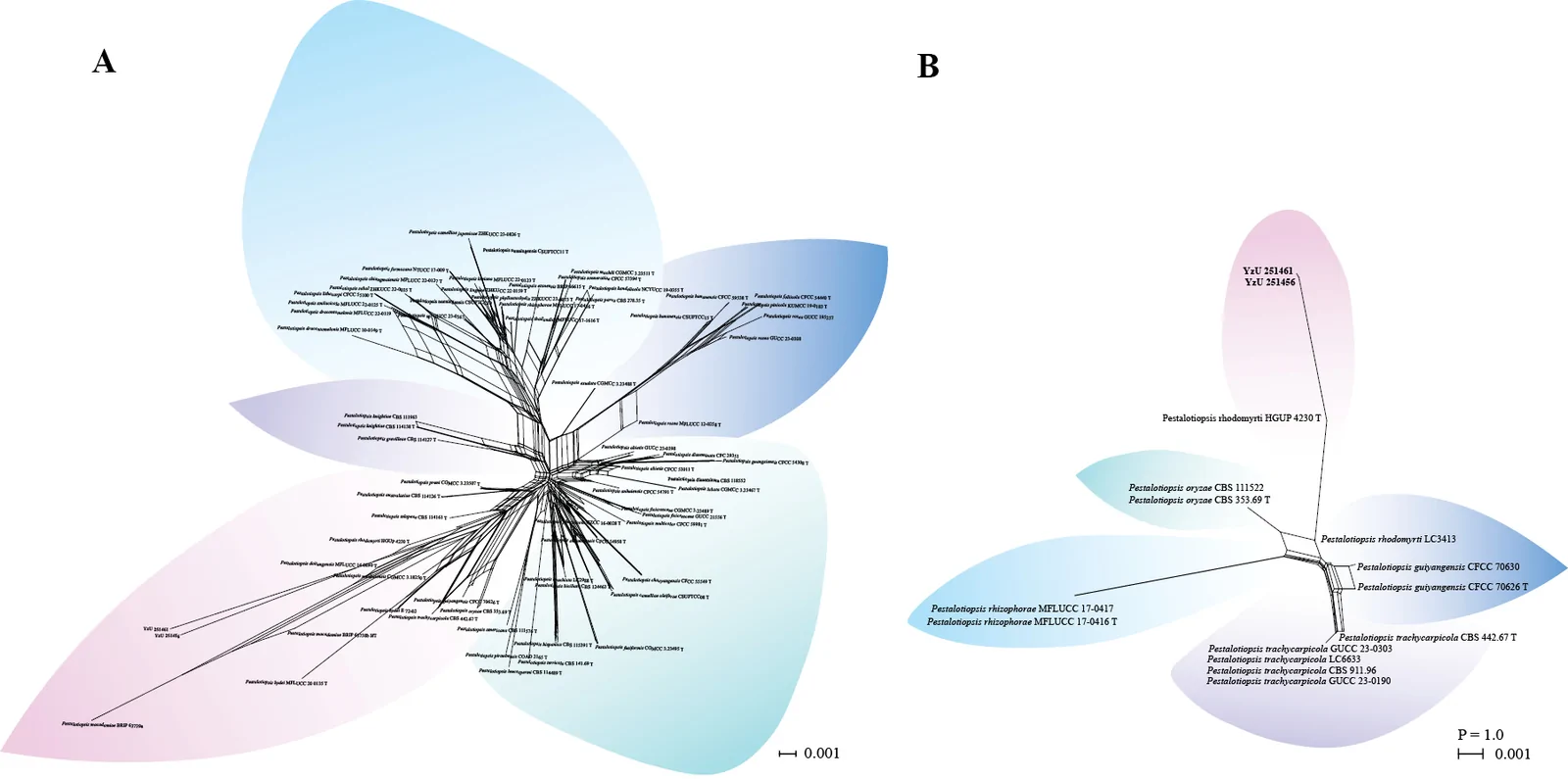
Neighbour-Net phylogenetic network of the genus *Pestalotiopsis*, based on the ITS, *TEF1* and *TUB2*. The network was constructed using LogDet transformation and the NeighbourNet algorithm implemented in SplitsTree4. **A**. Overall network showing the phylogenetic position of the new species; **B**. Enlarged view of the clade containing the new species and its closely-related congeners. Sequences generated in this study are shown in bold.

***Pestalotiopsis***: Multi-locus phylogeny (Total characters 1276: ITS = 541, *TEF1 =* 248, *TUB2* = 487) revealed a novel species (YzU 251456 and YzU 251461) in *Pestalotiopsis*. Best-fit models were as follows: GTR+I+G for ITS, *TUB2*; HKY+I+G for *TEF1* (Liu et al. 2017). According to the latest taxonomic framework proposed by Zhang et al. (2026), the genus *Pestalotiopsis* is divided into five species complexes. All strains examined in this study belong to the *Pestalotiopsis rosea* species complex (PRSC). Accordingly, phylogenetic trees were constructed using the PRSC (Fig. 6). The representative strains formed a stable, independent clade within this complex, with *P. rhodomyrti* (HGUP 4230T) identified as a closely-related species. This phylogenetic relationship was strongly supported (BS/PP = 99.3/0.85). In addition, no internal reticulation or interspecific gene flow showed as clear species boundaries between the two subclades, which indicated that the present two strains represented a new species (Fig. 7A, B).

### Species delimitation

**Figure 8.**
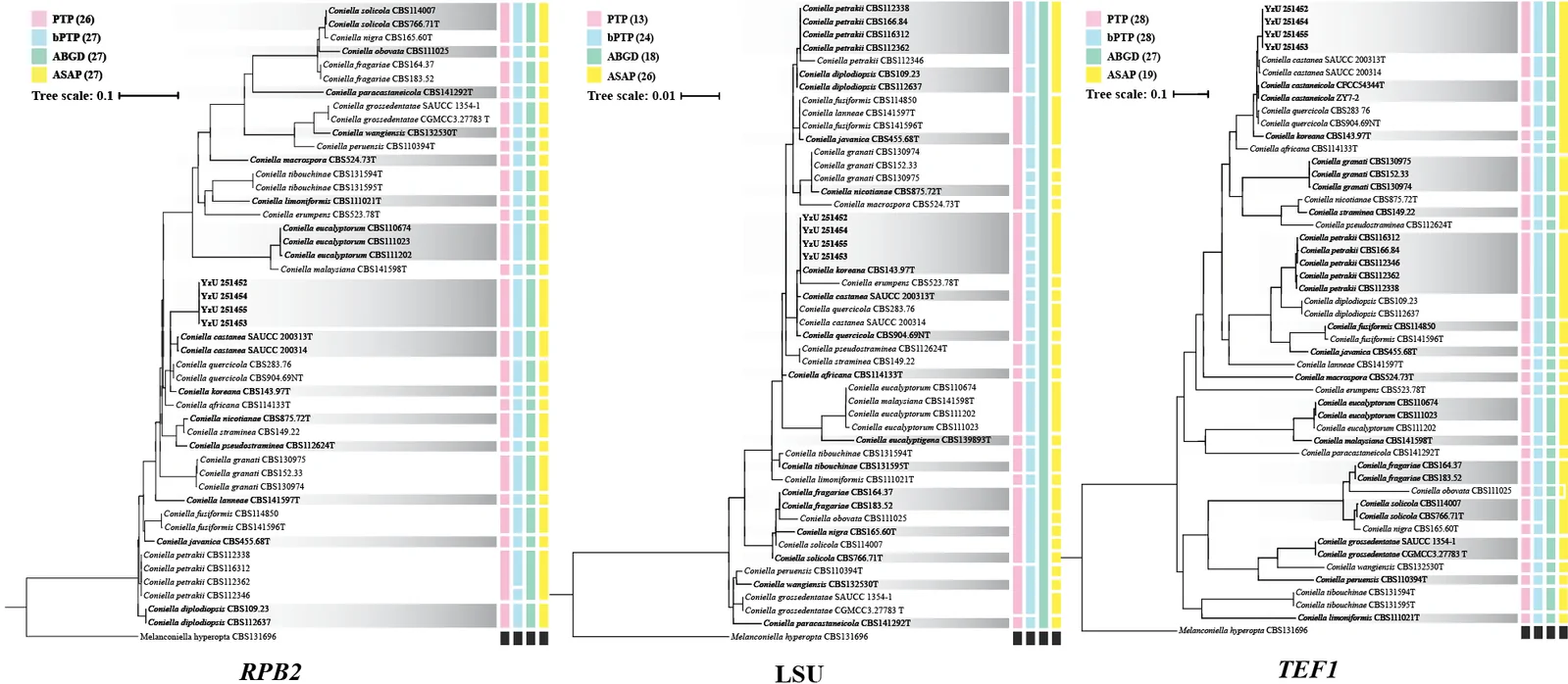
Maximum Likelihood (ML) single-gene phylogenetic trees of *Coniella* based on *TEF1*, *RPB2* and LSU sequences, showing species delimitation results under different criteria. Vertical coloured blocks correspond to different species delimitation methods: pink = PTP, light blue = bPTP, lake green = ABGD, yellow = ASAP. Numbers in parentheses in the legend indicate the total number of species delimited by each method. Isolates obtained in this study are shown in bold.

***Coniella***: GCPSR showed that *RPB2*, *TEF1* and LSU recovered the tested strains as a stable, independent lineage. *RPB2*/*TEF1* supported sister relationship to *C. castanea*, close relatedness by LSU. No significant recombination was detected at these three loci (PHI: P > 0.05). The ITS locus yielded insufficient informative sites for PHI analysis and lacked sufficient statistical reliability, thus was excluded from subsequent species delimitation analyses. The CBSR analyses implemented via PTP and bPTP algorithms showed clear species boundaries between the tested strains and *C. castanea* across all three loci. For DBSR analyses, ABGD under recursive partitions identified unambiguous barcode gaps for *RPB2* and *TEF1*, recovering 27 distinct species-level subsets and supporting our target strains as a novel entity. Although ABGD model for LSU merged the target isolates with closely-related congeners due to conservative sequence variation in this single locus, ASAP supported their distinct species status in *RPB2* (ASAP score = 3.5), but lumped the candidate strains with sister taxa in *TEF1* (score = 4.0) and LSU (score = 5.0), further reflecting the varying evolutionary resolution of individual barcode markers within *Coniella* (Fig. 8; Table 2).

**Table 2.** Multiple species recognition criteria were applied to single-locus and concatenated datasets to verify the target strain’s independent lineage for novel species validation. PHI test assessed recombination (complementary to GCPSR; p < 0.05 = significant recombination). ASAP post-slash number = confidence score (minimum 1; smaller = more robust delimitation).

**SRC**	**Method**	**Loci**							
		**ITS**	***RBP2***	***TEF1***	***LSU***	***TUB2***	***CHS***	***CAL***	***ACT***
*Coniella ampelopsidis* sp. nov.									
**GCPSR**	*PHI*	S/-	S/1.0	S/1.0	N/0.28				
**DBSR**	*ABGD*	-	S	S	N				
	*ASAP*/(score)	-	S/3.5	N/4.0	N/5.0				
**CBSR**	*PTP*	-	S	S	N				
	*bPTP*	-	S	S	S				
*Colletotrichum enshiense* sp. nov.									
**GCPSR**	*PHI*	N/-				S/1.0	N/-	S/1.0	S/1.0
**DBSR**	*ABGD*	-				S	-	N	S
	*ASAP*/(score)	-				N/6.0	-	S/6.0	N/3.5
**CBSR**	*PTP*	-				S	-	S	N
	*bPTP*	-				S	-	S	S
*Pestalotiopsis ampelopsidis* sp. nov.									
**GCPSR**	*PHI*	S/-		N/1.0		S/1.0			
**DBSR**	*ABGD*	-		N		N			
	*ASAP*/(score)	-		N/2.0		N/2.5			
**CBSR**	*PTP*	-		N		S			
	*bPTP*	-		S		S			

^1^ASAP = assemble species by automatic partitioning, ABGD = Automatic Barcode Gap Discovery, DBSR = distance-based species recognition, GCPSR = genealogical concordance phylogenetic species recognition, PHI = pairwise homoplasy index test, PTP = Poisson Tree Processes, bPTP = Bayesian PTP. ^2^ Abbreviations: S, support; N, no support. All species delimitation methods are independent, with support determined separately. Criteria: GCPSR, independent lineage in single-gene trees; DBSR: independent ASAP and ABGD analyses, each defined as distinct species delimitation; CBSR: independent PTP and bPTP analyses, each defined as a well-supported independent lineage in delimitation trees.

**Figure 9.**
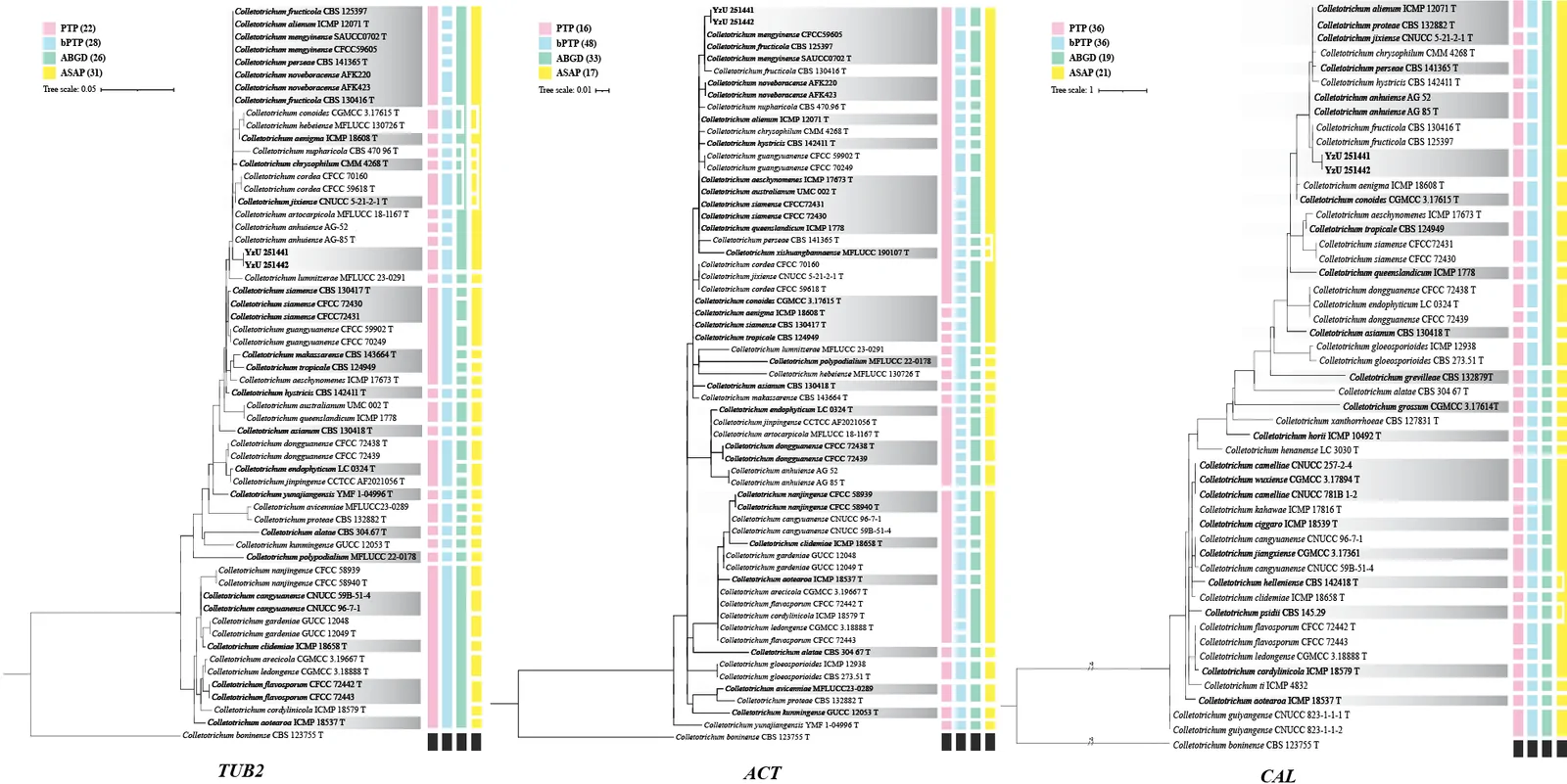
Maximum Likelihood (ML) single-gene phylogenetic trees of *Colletotrichum gloeosporioides* species complex, based on *ACT*, *CAL* and *TUB2* sequences, showing species delimitation results under different criteria. Vertical coloured blocks correspond to different species delimitation methods: pink = PTP, light blue = bPTP, lake green = ABGD, yellow = ASAP. Numbers in parentheses in the legend indicate the total number of species delimited by each method. Isolates obtained in this study are shown in bold.

***Colletotrichum***: GCPSR analyses revealed that strains YzU 251441 and 251442 were an independent evolutionary lineage by *ACT*, *CAL* and *TUB2*. No significant recombination was found by PHI tests (P > 0.05). ITS and *CHS* were excluded from subsequent analyses due to insufficient informative sites and low statistical reliability. CBSR analyses by PTP and bPTP algorithms had clear species boundaries between the two isolates and *Col. mengyinense* across all three loci. For DBSR analyses, ABGD under recursive partitions identified unambiguous barcode gaps for *ACT*, recovering 34 distinct species-level subsets and supporting our target strains as a novel entity. Although ABGD models for *CAL* and *TUB2* merged the target isolates with closely-related congeners due to conservative sequence variation in these single loci, ASAP lumped the candidate strains with closely-related congeners (including *Col. fructicola* and *Col. mengyinense*) across the evaluated single-locus alignments (*ACT*, *CAL* and *TUB2*), further reflecting the highly conservative nature of individual barcode markers within the *Colletotrichum gloeosporioides* species complex (Fig. 9; Table 2).

**Figure 10.**
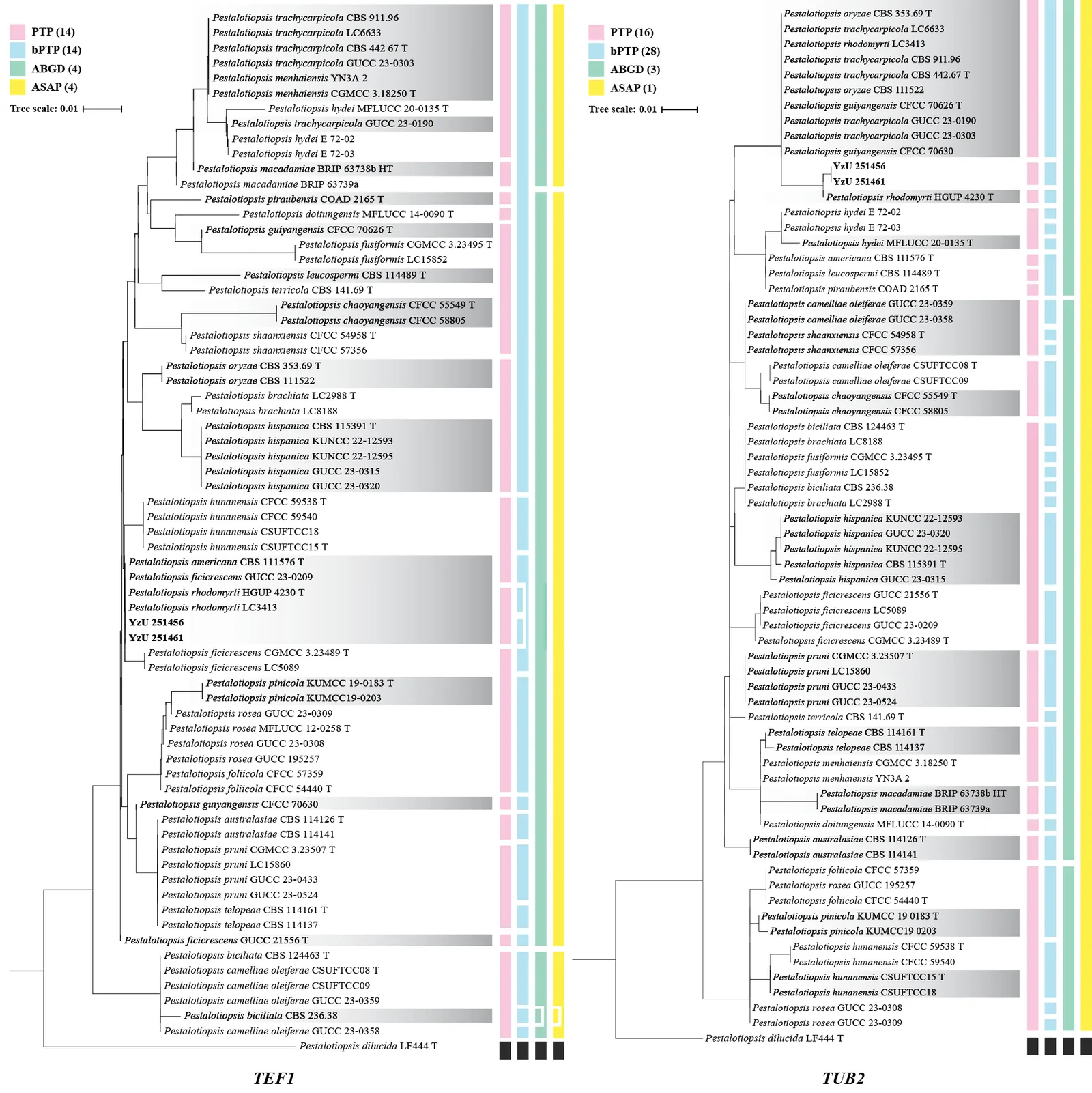
Maximum Likelihood (ML) single-gene phylogenetic trees of *Pestalotiopsis*, based on *TEF1* and *TUB2* sequences, showing species delimitation results under different criteria. Vertical coloured blocks correspond to different species delimitation methods: pink = PTP, light blue = bPTP, lake green = ABGD, yellow = ASAP. Numbers in parentheses in the legend indicate the total number of species delimited by each method. Isolates obtained in this study are shown in bold.

***Pestalotiopsis***: The ITS region was excluded from further analyses owing to limited informative sites and poor statistical support in PHI tests. GCPSR analyses indicated that strains YzU 251456 and YzU 251461 constitute a separate evolutionary lineage in the *TUB2*, while the *TEF1* phylogeny was unresolved. No significant recombination was detected between this lineage and its close relatives via PHI tests (PHI, *P* > 0.05). For CBSR analyses, both PTP and bPTP recovered clear species boundaries between the two isolates and allied taxa using *TUB2*, whereas only bPTP supported such boundaries with *TEF1*. In DBSR analyses, recursive ABGD partitions grouped the target isolates with closely-related species for both *TEF1* and *TUB2*; ASAP analyses failed to resolve species boundaries within this *Pestalotiopsis* complex, indiscriminately merging the candidate strains along with multiple recognised neighbouring taxa into broad clusters across all single-locus alignments (*TEF1* score = 2.0; *TUB2* score = 2.5). These findings highlight the high sequence conservation of individual barcode markers within *Pestalotiopsis* (Fig. 10; Table 2).

### SNP principal component analysis

**Figure 11.**
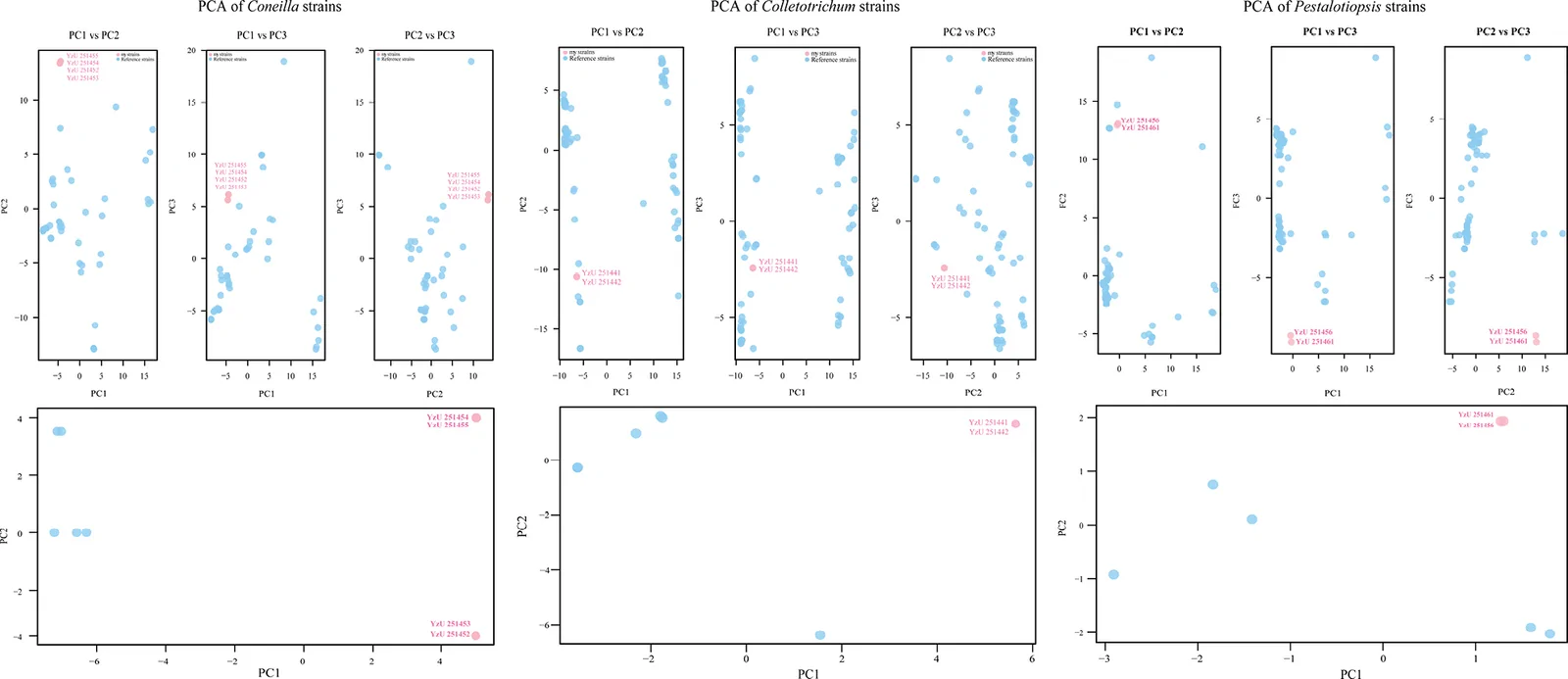
PCA based on SNPs extracted from concatenated multi-locus alignments of *Coniella*, *Colletotrichum* and *Pestalotiopsis* strains. Blue circles represent reference strains and pink circles represent our isolates. The upper panels show pairwise projections of PC1 vs. PC2, PC1 vs. PC3 and PC2 vs. PC3 for each genus. The lower enlarged panels display PCA plots of the target isolates and their closest relatives, highlighting the genetic separation between our isolates and the corresponding reference taxa.

Independent SNP-based PCA of concatenated multi-locus alignments for *Coniella*, *Colletotrichum* and *Pestalotiopsis* consistently revealed significant genetic differentiation between the present strains and their closely-related species. All tested strains formed distinct, well-separated clusters in PCA plots and these findings are fully concordant with our multi-locus phylogenetic and species delimitation results (Fig. 11).

### Taxonomy

#### Coniella ampelopsidis

Taxon classificationFungiDiaporthalesSchizoparmaceae

S.Q. Chen & J.X. Deng sp. nov.

216473C8-4E45-5CC5-A3A8-BEE362165F98

863685

Fig. 12

##### Etymology.

The epithet refers to the host plant from which the holotype was isolated.

**Figure 12.**
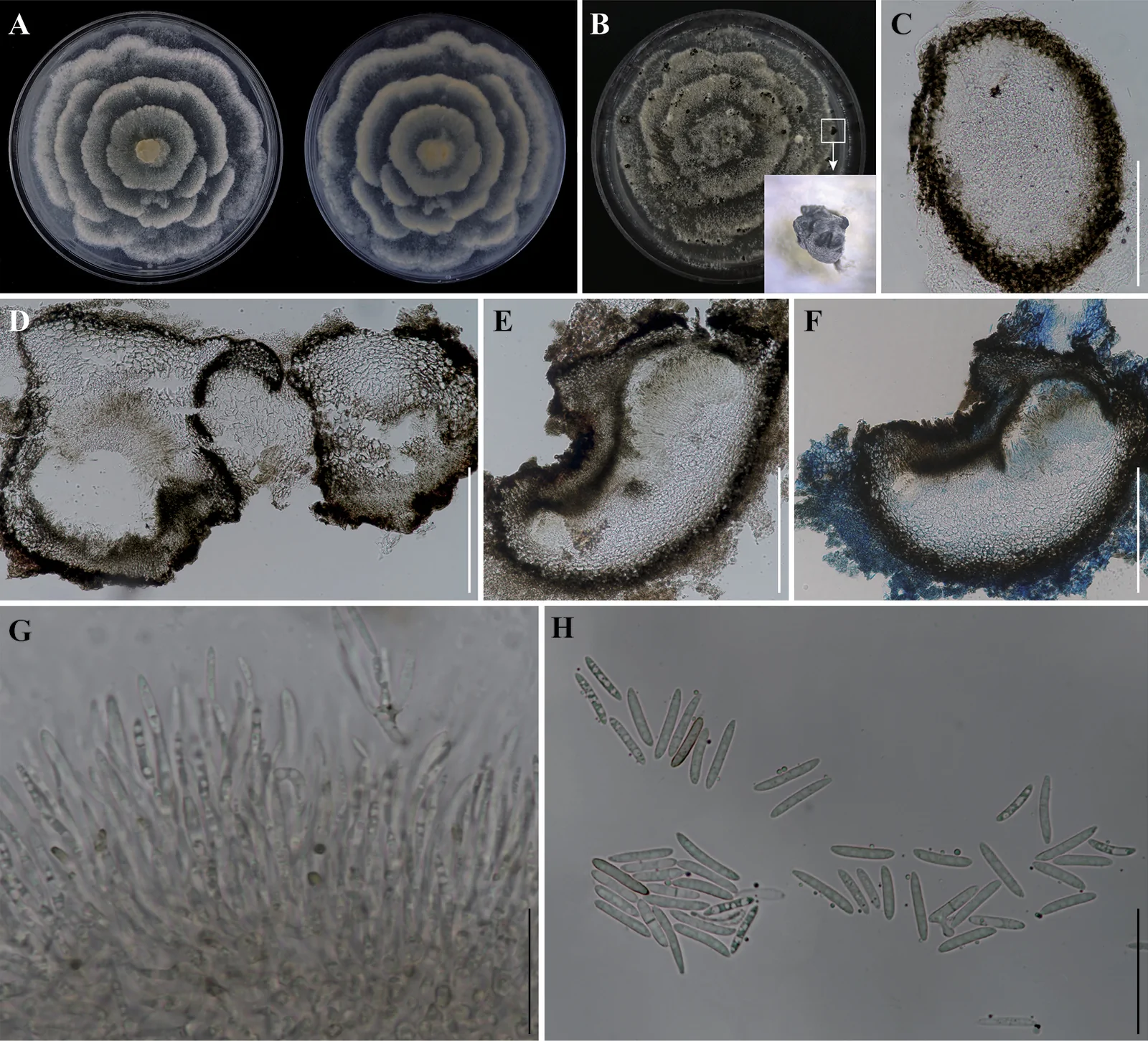
Morphological characteristics of *Coniella ampelopsidis* sp. nov. (extype YzU 251452). **A**. Colony on PDA after 7 days at 25 °C; **B**. Colony showing conidiomata formation, inset: single conidioma; **C**. Developing conidiomata; **D–F**. Cross-sections of conidiomata; **G**. Conidiogenous cells; **H**. Conidia. Scale bars: 200 µm (**C–F**); 25 µm (**G, H**).

##### Typification.

China, • Hubei Province, Enshi City (30°19'46"N, 109°32'19"E), from leaf disease of *Nekemias grossedentata*. June 2025, S.Q. Chen, ex-type culture YzU 251452.

##### Description.

***Colonies*** on PDA (25 °C, dark) after 7 d reaching 78–80 mm. Circular, raised, undulate margin, velvety, radial and petaloid growth pattern; aerial mycelium sparse, forming distinct concentric rings, white throughout; reverse concolorous with the obverse. ***Conidiomata*** pycnidial, solitary to gregarious, immersed to erumpent, globose to subglobose, dark brown to black, unilocular, ostiolate. ***Conidiophores*** reduced to conidiogenous cells, arising directly from the inner layer of the pycnidial wall. ***Conidiogenous cells*** phialidic, ampulliform to lageniform, hyaline, smooth-walled, enteroblastic, monophialidic. ***Conidia*** unicellular, hyaline, smooth-walled, leguminiform, oblong, containing 4–8 guttules, (12.6–)17.5–20.9(–21.4) × (1.63–)2.14–2.53(–2.89) μm (av.: 17.28 ± 1.65 × 2.32 ± 0.25 μm; L/W = 7.54).

##### Notes.

Conidia of *Coniella* are divided into two morphological groups: olivaceous brown to brown, ellipsoid to globose; and hyaline, fusiform to clavate (Crous et al. 2014). *C. ampelopsidis* morphologically resembles *C. castanea* and *C. quercicola* in producing cylindrical to leguminiform, occasionally fusiform, navicular or clavate conidia. Morphologically, *C. ampelopsidis* can be clearly distinguished from these congeners primarily by its significantly narrower conidia, higher length/width (L/W) ratio and distinct conidial shape (Table 3). Phylogenetic analyses placed *C. ampelopsidis* and *C. castanea* in the same major clade. Species delimitation analyses recovered clear interspecific boundaries. SNP-PCA visualised their nucleotide divergence. Multi-locus phylogenetic analyses provide stable and accurate interspecific resolution within *Coniella*, which, together with species delimitation, corroborates *C. ampelopsidis* as a distinct new species.

**Table 3.** Morphological characteristics and host information of *Coniella ampelopsidis* and related *Coniella* species.

**Species**	**Conidial color and shape**	**Conidial size (μm)**	**Conidial L/W ratio**	**Host / Origin**	**Reference**
***Coniella ampelopsidis***	**Hyaline, leguminiform to oblong**	**17.5–20.9** × **2.14–2.53**	**7.5**	***Nekemias grossedentata* / Hubei, China**	**Present study**
*Coniella castanea*	Pale yellowish-brown, straight or sinuous	14.20–22.92 × 2.51–4.11	6.3	*Castanea mollissima* / Shandong, China	Wang et al. (2022)
*Coniella quercicola*	Hyaline, cylindrical, slightly curved to naviculate	14–18 × 2.5–3	5.3	*Quercus robur* / The Netherlands	Alvarez et al. (2016)
*Coniella africana*	Hyaline to pale yellowish, linear, cylindrical to naviculate	15–20.5 × 3-3.5	6.0	*Eucalyptus* sp. / South Africa	
*Coniella koreana*	Hyaline to pale yellowish-brown, cylindrical, curved to falcate	16–19 × 2.5–3	6.5	*Vitis vinifera* / South Korea	

#### Colletotrichum enshiense

Taxon classificationFungiGlomerellalesGlomerellaceae

S.Q. Chen & J.X. Deng sp. nov.

3EDF3B24-C36B-5691-8136-065E352A9CEA

863687

Fig. 13

##### Etymology.

The specific epithet refers to the locality from which the holotype specimen was obtained.

**Figure 13.**
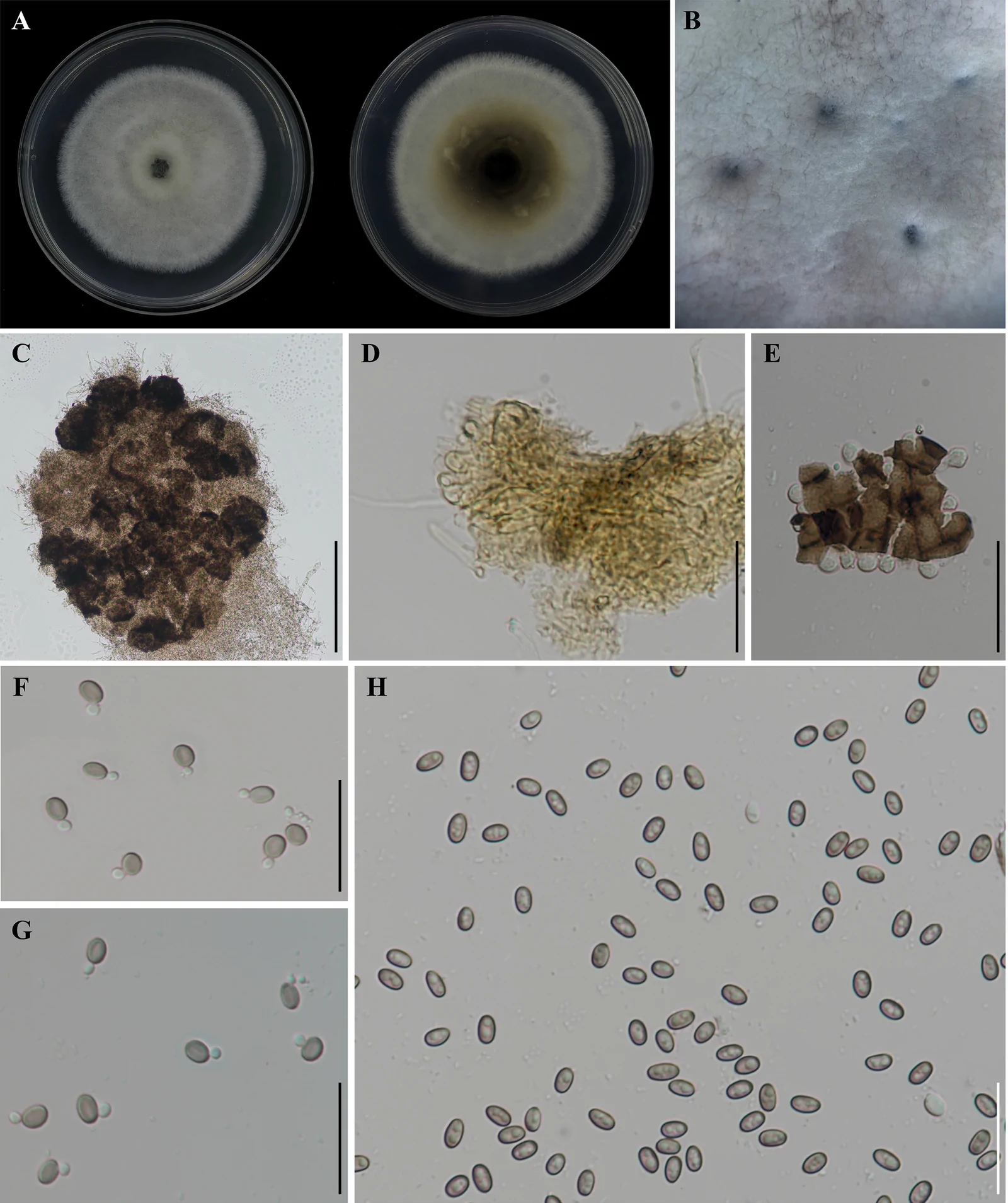
Morphological characteristics of *Colletotrichum enshiense* sp. nov. (extype YzU 251441). **A**. Colonies on PDA after 7 days at 25 °C; **B, C**. Acervuli; **D**. Conidiogenous layer of the acervulus; **E**. Acervular tissue; **F, G**. Germinating conidia; **H**. Conidia. Scale bars: 150 μm (**C**); 15 μm (**H**); 20 μm (**D–G**).

##### Typification.

China, • Hubei Province, Enshi City (30°19'50"N, 109°32'07"E), from leaf disease of *Nekemias grossedentata*. June 2025, H. Luo, ex-type culture YzU 251441.

##### Description.

***Colonies*** on PDA (25 °C, in darkness after 7 d) reaching 63–65 mm. Circular, umbonate, with an entire margin; aerial mycelium dense, cottony or villous, creamy-white to white; reverse margin white; yellowish-brown at the margin, becoming dark brown to black at the centre. ***Conidiomata*** acervular, solitary to gregarious, dark brown to black, setae absent. ***Conidiophores*** directly formed on hyphae and usually degenerate into conidiogenous cells. ***Conidia*** unicellular, hyaline, smooth-walled, ellipsoidal to ovoid, both ends rounded, (4.2–)4.8–5.7(–6.1) × (2.1–)2.7–3.3(–3.8) μm (av.: 5.15 ± 0.38 c 3.04 ± 0.25 μm), with 2–4 granular contents. Appressoria not observed under the tested culture conditions.

##### Notes.

*Colletotrichum enshiense* is phylogenetically placed within the *Colletotrichum gloeosporioides* species complex (CGSC) and forms a well-supported clade closely related to *Col. fructicola* and *Col. mengyinense*. Morphologically, *Col. enshiense* is readily distinguished from these phylogenetically closely-related congeners by its remarkably small, compact, ellipsoidal to ovoid conidia (4.8–5.7 × 2.7–3.3 μm; av.: 5.15 × 3.04 μm) (Table 4). Although appressoria were not observed under the tested culture conditions, *Col. enshiense* differs distinctly from *Col. fructicola* and other congeners, which typically produce much larger, elongated cylindrical conidia (> 11.0 μm in length). Overall, *Col. enshiense* is unambiguously differentiated from close relatives by fixed diagnostic nucleotide substitutions and distinct SNP-based PCA clustering. Although single-locus distance-based methods (ABGD and ASAP) show limited resolution due to conservative sequence variation, the independent species status of *Col. enshiense* is fully corroborated by multi-locus concatenated phylogeny (ML/BI), concordant coalescent models (PTP/bPTP) and distinct morphology.

**Table 4.** Morphological characteristics and host information of *Colletotrichum enshiense* and related *Colletotrichum* species.

**Species**	**Conidial shape**	**Conidial size (μm)**	**Appressoria size and shape (μm**)	**Host/Origin**	**Reference**
***Colletotrichum enshiense***	**Ellipsoidal to ovoid, ends rounded**	**4.8–5.7 × 2.7–3.3**	**not observed**	***Nekemias grossedentata* / Hubei, China**	**Present study**
*Colletotrichum fructicola*	hyaline, cylindrical, with obtuse to rounded ends	9.7–14 × 3–4.3	6.0–14.5 × 4.0–9.5, ovate to clavate, occasionally irregular or slightly lobed	*Coffea arabica* / Thailand	Prihastuti et al. (2009)
*Colletotrichum mengyinense*	Cylindrical to clavate, ends rounded	13.0–17.5 × 4.0–6.0	7.5–13.5 × 5.0–9.0, subglobose to ellipsoid, medium brown to dark brown	*Prunus persica* / Shandong, China	Liu et al. (2022)
*Colletotrichum noveboracense*	Cylindrical, ends rounded	13.5–16.5 × 5.0–6.0	6.5–12.0 × 5.0–8.5, subglobose to irregular, medium brown	*Malus domestica* / USA	Wallhead et al. (2014)
*Colletotrichum chrysophilum*	Cylindrical, both ends rounded	13.0–16.5 × 4.5–5.5	8.0–14.0 × 5.5–9.0, medium brown to dark brown, clavate, irregular or lobed	*Musa* sp. / Brazil	Damm et al. (2012)
*Colletotrichum corde*a	Cylindrical to oblong	12.5–17.0 × 4.5–6.0	6.5–11.5 × 4.5–8.0, subglobose to ellipsoidal, medium brown to dark brown	*Juglans regia* / China	Li et al. (2024)
*Colletotrichum jixiense*	Cylindrical, straight, both ends rounded	13.0–18.5 × 4.0–5.5	7.0–12.5 × 5.0–8.5, ellipsoidal to irregular, often lobed, medium brown to dark brown	*Glycine max* / Heilongjiang, China	Liu et al. (2022)

#### Pestalotiopsis ampelopsidis

Taxon classificationFungiAmphisphaerialesPestalotiopsidaceae

S.Q. Chen & J.X. Deng sp. nov.

98F33C49-51E5-5E1E-956B-D9A2186DE1D8

863688

Fig. 14

##### Etymology.

The epithet refers to the host plant from which the holotype was isolated.

**Figure 14.**
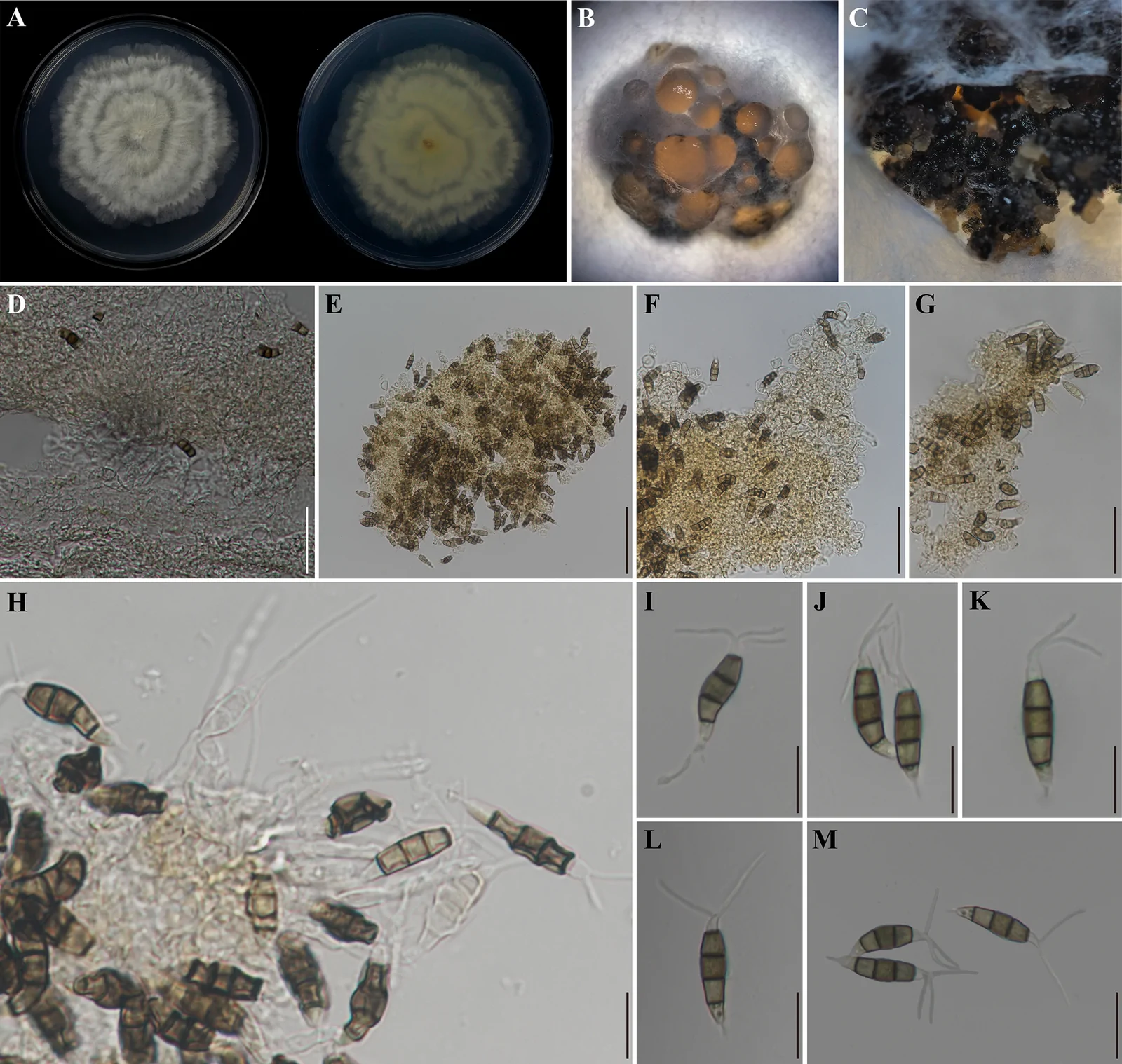
Morphological characteristics of *Pestalotiopsis ampelopsidis* sp. nov. (extype YzU 251456). **A**. Colonies on PDA after 7 days at 25 °C; **B**. Acervuli formed on PDA; **C**. Close-up view of acervuli; **D**. Cross-sections of acervuli; **E–G**. Conidial masses extruded from acervuli and conidiogenous cells; **H**. Mode of conidiogenesis; **I–M**. Conidia. Scale bars: 60 μm (**D, E**); 30 μm (**F, G**); 10 μm (**H–M**).

##### Typification.

China, • Hubei Province, Enshi City (30°19'46"N, 109°32'19"E), from leaf disease of *Nekemias grossedentata*. June, 2025, Q.H. Chen, ex-type culture YzU 251456.

##### Description.

Colonies on PDA (25 °C, 7 d, in darkness) reaching 60–65 mm, irregularly circular, flat, irregular margin; aerial mycelium moderate, floccose to cottony, off-white, distinct concentric rings, radial growth pattern; reverse pale yellow. ***Conidiomata*** saucer-shaped, scattered or gregarious, superficial to immersed, shining, exuding black conidial masses. ***Conidiophores*** hyaline, septate, branched at the base, smooth-walled. ***Conidiogenous cells*** phialidic, cylindrical, hyaline, solitary to aggregated. ***Conidia*** fusiform to ellipsoidal, smooth-walled, slightly constricted at septa, with 3–4 septa, (16.6–)19.5–25.0(–26.38) × (4.8–)5.3–6.9(–8.1) μm (av.: 22.50 ± 2.5 × 6.22 ± 0.77 μm) and the basal cell hyaline, obconical, 2.5–4.6 μm long. Three median cells barrel-shaped, verrucose, uniformly brown. Apical cell hyaline, smooth-walled, conical to trapezoidal, tapering towards the apex, 2.1–4.2 μm long. Bearing 2–3 (mostly 2, rarely 3) apical tubular appendages, arising from the apical protuberance, unbranched, filiform, 7.2–13.6 μm long. A single central basal tubular appendage present at the base, unbranched, 2.3–4.5 μm long.

##### Notes.

Morphological characters, such as conidial dimensions, provide limited resolution for species delimitation in *Pestalotiopsis* (Maharachchikumbura et al. 2014). Despite the inherent morphological conservatism in *Pestalotiopsis*, *P. ampelopsidis* can still be distinguished from these closely-related congeners by a unique combination of conidial dimensions and apical/basal appendage characteristics (Table 5). Phylogenetically, according to the taxonomic framework proposed by Zhang et al. (2026), *Pestalotiopsis ampelopsidis* belongs to the *Pestalotiopsis rosea* species complex (PRSC). It forms a well-supported monophyletic lineage closely related to *P. rhodomyrti*, *P. oryzae*, *P. trachycarpicola* and *P. guiyangensis*. Although DBSR completely failed to resolve species boundaries due to high sequence conservation and the absence of clear barcode gaps, CBSR and GCPSR firmly established its distinct species boundary, which is further corroborated by SNP-based PCA. Conclusively, the integration of multi-locus phylogenetic frameworks, coalescent delimitation and genomic data validates *P. ampelopsidis* as a distinct novel entity.

**Table 5.** Morphological characteristics and host information of *Pestalotiopsis ampelopsidis* and related *Pestalotiopsis* species.

**Species**	**Conidial size (μm)**	**Median cells characteristics**	**Apical appendages (No. and length, μm)**	**Basal appendage length (μm)**	**Host/Origin**	**Reference**
***Pestalotiopsis ampelopsidis***	**19.5–25.0 × 5.3–6.9**	**Barrel-shaped, verrucose, concolorous brown**	**2–3 (mostly 2), 7.2–13.6**	**2.3–4.5**	***Nekemias grossedentata* / Hubei, China**	**Present study**
*Pestalotiopsis rhodomyrti*	20.0–27.0 × 4.9–8.0	Barrel-shaped (doliiform) to subcylindrical, concolorous brown	2–3, 7.5–17.5	2.0–7.0	*Rhodomyrtus tomentosa* / Guangxi, China	Zhang et al. (2012)
*Pestalotiopsis oryzae*	23.5–32.0 × 5.0–7.0	Versicolorous; upper cells pale brown, lower cell reddish-brown	2–4, 12.0–25.0	3.0–8.5	*Oryza sativa* / India	Maharachchikumbura et al. (2014)
*Pestalotiopsis trachycarpicola*	18.0–24.0 × 6.5–8.5	Barrel-shaped (doliiform), concolorous dark brown	3–5 (mostly 3–4), 15.0–26.0	4.0–9.0	*Trachycarpus fortunei* / Zhejiang, China	Zhang et al. (2013)
*Pestalotiopsis guiyangensis*	19.0–26.5 × 5.5–7.5	Barrel-shaped, concolorous dark brown	2–3, 8.5–18.0	3.0–6.0	*Camellia sinensis* / Guizhou, China	Liu et al. (2017)

### Pathogenicity test

**Figure 15.**
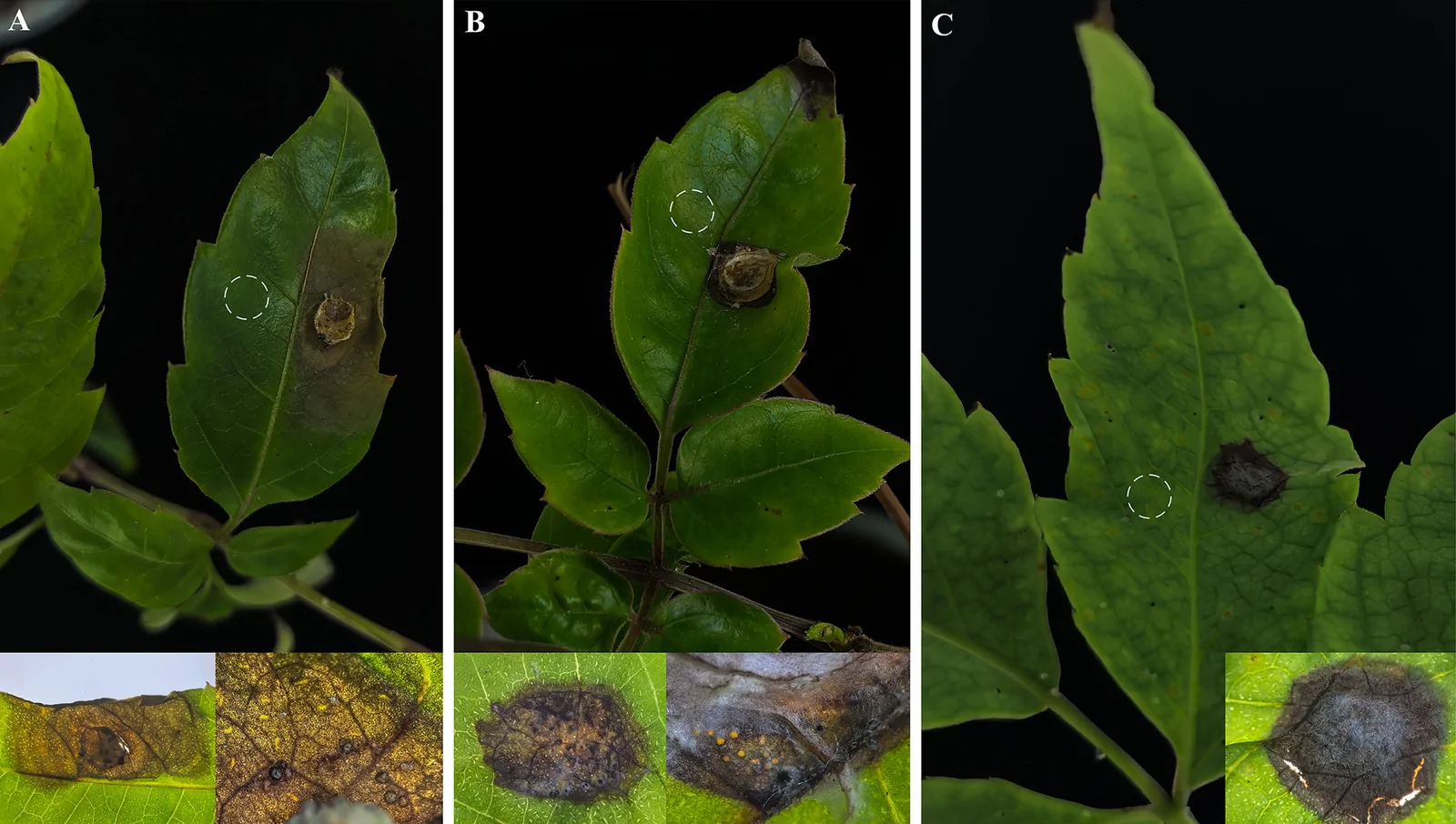
Pathogenicity test results of the three fungal species on living leaves of *Nekemias grossedentata* at 7 days’ post-inoculation. **A**. Necrotic lesion caused by *Coniella ampelopsidis*; **B**. Necrotic lesion caused by *Pestalotiopsis ampelopsidis*.; **C**. Necrotic lesion caused by *Colletotrichum enshiense*. Sterile PDA plugs were used as the control group. The area outlined by the white dotted circle indicates the inoculation site of the control and no symptoms were observed.

**Figure 16.**
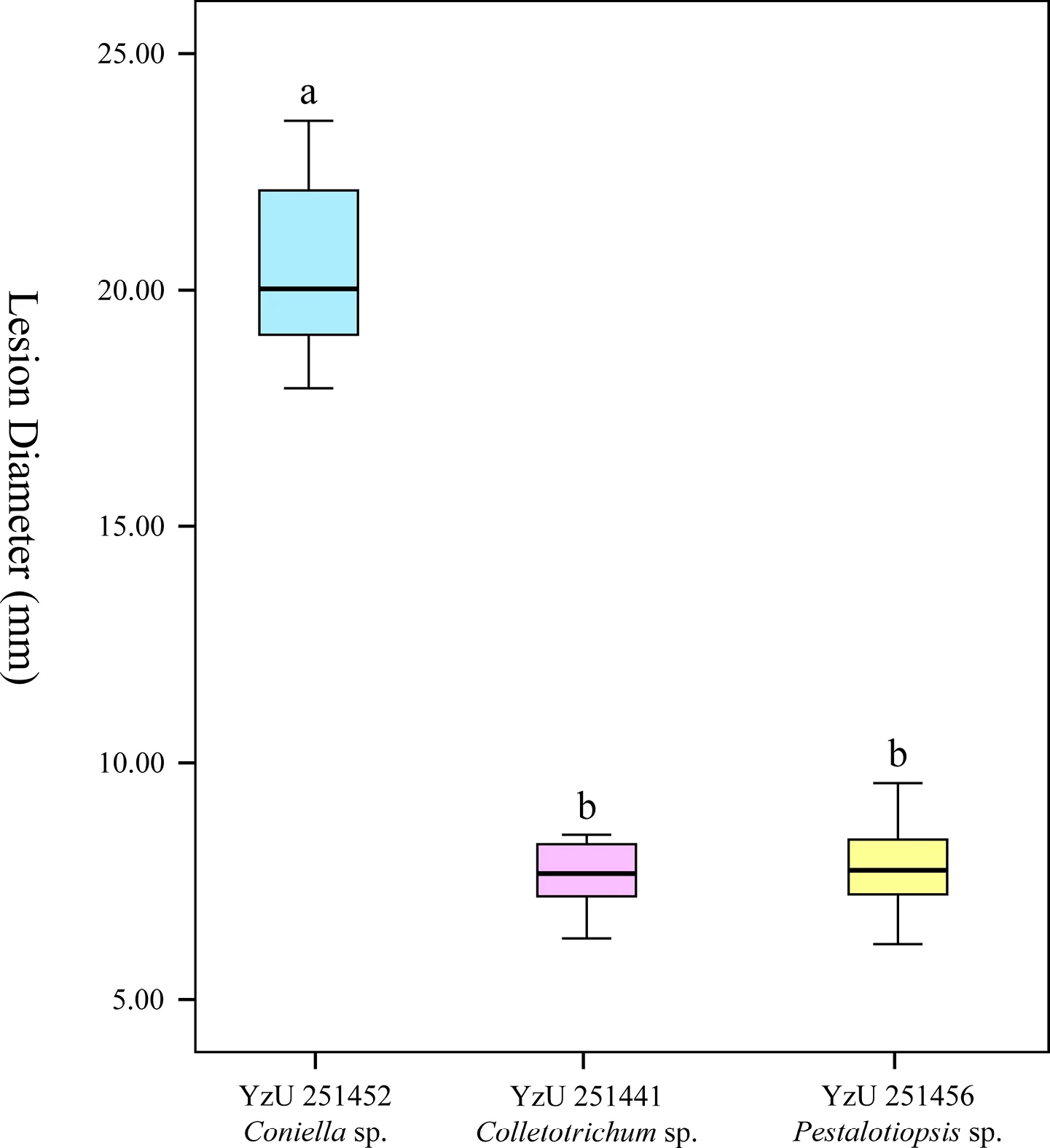
Box plot showing lesion size (mm) caused by the present strains on *Nekemias grossedentata*. Different lowercase letters (**a, b, c**) indicate significant differences in pathogenicity amongst strains at p < 0.05 (one-way ANOVA, followed by Tukey’s test).

Pathogenicity assays showed that *Coniella ampelopsidis*, *Colletotrichum enshiense* and *Pestalotiopsis ampelopsidis* were all pathogenic to leaves of *Nekemias grossedentata*, with significant differences in virulence (Figs 15, 16). *C. ampelopsidis* exhibited the strongest virulence, causing irregular yellowish-brown lesions with an average diameter of 20.8 ± 0.32 mm, which spread rapidly and resulted in leaf necrosis. No significant difference in virulence was observed between *P. ampelopsidis* and *Col. enshiense*, which induced distinct leaf spot symptoms with average lesion diameters of 9.8 ± 0.10 mm and 10.2 ± 0.17 mm, respectively (Fig. 16). Lesions caused by *C. ampelopsidis* were yellowish-brown and irregularly spreading. Distinct brown to black pycnidia were borne on the leaf surface within lesions. *P. ampelopsidis* and *Col. enshiense* caused dark brown, circular lesions. Distinct black pycnidia were visible within lesions produced by *P. ampelopsidis*.

## Discussion

*Coniella (Schizoparmaceae)* is a cosmopolitan genus occurring as plant pathogens, endophytes and saprobes on diverse hosts. Its taxonomy has been substantially revised in recent years, multi-locus phylogeny showed *Pilidiella* and *Schizoparme* are congeneric with *Coniella* and have been synonymised under the latter (Alvarez et al. 2016). As pathogens, *Coniella granati* causes pomegranate fruit rot and twig blight in Anhui (Chen et al. 2014); *C. vitis* causes grapevine white rot across multiple Chinese regions (Chethana et al. 2017); *C. castanea* was described from diseased *Fagaceae* leaves in a Shandong orchard (Wang et al. 2022). Four novel *Coniella* species were described from different host plants in Wuyishan, Fujian, of which *C. grossedentatae* was a new species from *Nekemias grossedentata* leaves with untested pathogenicity (Li et al. 2025).

*Colletotrichum* (*Glomerellales*, *Glomerellaceae*) is a major genus of plant pathogens affecting numerous economically important crops worldwide (Hyde et al. 2009). Its taxonomy has been substantially reshaped by molecular phylogenetics; it now comprises ~ 340–350 species grouped into 15–20 species complexes across different classification frameworks (Talhinhas and Baroncelli 2021; Liu et al. 2022; Talhinhas and Baroncelli 2023; Usman et al. 2026). In recent years, the ApMat locus has been increasingly adopted as an additional phylogenetic marker, providing improved resolution for species delimitation, particularly within the *Colletotrichum gloeosporioides* species complex (CGSC) (Silva et al. 2012; Vieira et al. 2020). The CGSC is the largest and most polyphagous lineage in *Colletotrichum*, comprising numerous phytopathogenic species infecting a wide range of host plants, including many economically important members of the *Vitaceae* (Weir et al. 2012).

*Pestalotiopsis* (*Amphisphaeriales*, *Sporocadaceae*) is a cosmopolitan genus with a broad host range (Maharachchikumbura et al. 2014). Species occur as plant pathogens, saprophytes and endophytes (Maharachchikumbura et al. 2011). As pathogens, it causes diverse plant diseases, *P. camelliae* inducing camellia leaf blight (Zhang et al. 2012); *P. neglecta* causing black spot disease on Pinus sylvestris var. mongolica (Chen et al. 2020); *P. jiangsuensis* infecting *Pinus massoniana* and resulting needle greying and blight (Li et al. 2024); *P. oryzae* causing leaf spot on *Lanxangia tsaoko* (Zhao et al. 2025). Species in this genus have an extremely broad host range spanning more than 80 plant families worldwide (Maharachchikumbura et al. 2014). There is no report formally infecting plants from the genus *Ampelopsis*. In the present study, *P. ampelopsidis* sp. nov. was isolated from *N. grossedentata* as the causal agent of leaf spot disease, which expands the known host range of *Pestalotiopsis*.

Phylogenetic patterns help clarify the evolutionary origins of these foliar pathogens. The closest relatives of *Coniella ampelopsidis* (*C. castanea* and *C. quercicola*) are primary pathogens on *Fagaceae* hosts (Jiang et al. 2021; Jankowiak et al. 2022; Wang et al. 2022). As *Fagaceae* species form the dominant forest canopy in the mountainous ecosystems of Enshi Prefecture, spatial overlap and shared microclimates between oak forests and vine tea plantations likely facilitated ecological contact. In phytopathogenic fungi, sympatric proximity frequently provides opportunities for host jumping or host shifts, driving adaptive divergence and speciation (Damien et al. 2013; Thines 2019). Similarly, *Colletotrichum enshiense* and *Pestalotiopsis ampelopsidis* are sister to broad-host-range pathogens (*Col. fructicola* and *P. oryzae*, respectively), suggesting that ecological spill-over and host adaptation may have driven their lineage separation. SNP-based PCA and multi-locus species delimitation analyses demonstrate distinct genetic isolation between these novel isolates and their close relatives, supporting their status as independent evolutionary lineages. Although confirming host shifts requires further cross-inoculation assays, ancestral state reconstructions and comparative genomics targeting effector-driven host specificity (Dong et al. 2015; Baroncelli et al. 2016), current findings support a plausible host-shift hypothesis. From an epidemiological perspective, origin via cross-host transmission from surrounding forest vegetation indicates that integrated disease management of vine tea should extend beyond field sanitation to incorporate surveillance of adjacent wild vegetation.

Integrative taxonomy combining morphology, multi-locus phylogeny and coalescent species delimitation provides a robust framework for resolving cryptic fungal species (Taylor et al. 2000; Lücking et al. 2020; Hyde et al. 2024). While traditional conidial morphology failed to resolve species boundaries due to phenotypic overlap, coalescent algorithms (PTP, bPTP, ABGD, ASAP) successfully validated genetic discontinuities defining independent lineages (Carstens et al. 2013; Dissanayake et al. 2024). Additionally, SNP-based PCA extracted from concatenated sequences resolved fine-scale nucleotide differentiation, unambiguously separating the three novel species from their closest relatives. Future integration of this approach with phylogenomics and phenomics will further refine fungal species delimitation and advance pathogen evolutionary insights (Tekpinar and Kalmer 2022; Hyde et al. 2024).

## Conclusions

In this study, three novel foliar pathogenic fungal species—*Coniella ampelopsidis*, *Colletotrichum enshiense*, and *Pestalotiopsis ampelopsidis*—were formally identified and described from diseased *Nekemias grossedentata* leaves in Hubei, China. An integrative taxonomic approach combining morphological characterisation, multi-locus phylogeny, coalescent species delimitation algorithms and SNP-based PCA consistently delimited their distinct species boundaries. Pathogenicity assays fulfilled Koch’s postulates and revealed significant virulence variation amongst the three species, with *C. ampelopsidis* exhibiting the greatest aggressive behaviour. These findings document three phylogenetically distant fungal pathogens from three distinct orders co-occurring on a single host, expanding the known disease spectrum of *N. grossedentata*. Overall, this study demonstrates the effectiveness of an integrative taxonomic framework in resolving cryptic fungal pathogens on economic crops and provides a scientific foundation for regional disease monitoring and integrated management of vine tea.

## Supplementary Material

XML Treatment for Coniella ampelopsidis

XML Treatment for Colletotrichum enshiense

XML Treatment for Pestalotiopsis ampelopsidis

## Additional information

### Artificial Intelligence (AI) use

The authors accept full responsibility for the content of the manuscript, including the disclosure of any use of AI.

No AI tools were used in the preparation of this manuscript.

### Author ORCIDs

S.-Q. Chen https://orcid.org/0009-0003-7533-921X

X.-T. Zhou https://orcid.org/0009-0006-1346-2337

X. Mou https://orcid.org/0009-0004-8275-2739

S.-X. Zhou https://orcid.org/0009-0000-1581-6924

Q. Deng https://orcid.org/0009-0000-4097-1520

J.-X. Deng https://orcid.org/0000-0001-7304-5603

H.-F. Liu https://orcid.org/0000-0002-9733-9240

### Data availability

All of the data that support the findings of this study are available in the main text or Supplementary Information.

## Supplementary materials

Supplementary material 1 Strains used in this study and their GenBank accession numbers Data type pdf https://binary.pensoft.net/file/1769861This dataset is made available under the Open Database License (http://opendatacommons.org/licenses/odbl/1.0/). The Open Database License (ODbL) is a license agreement intended to allow users to freely share, modify, and use this Dataset while maintaining this same freedom for others, provided that the original source and author(s) are credited. Shi-Qiang Chen,

## References

[B1] Alvarez LV, Groenewald JZ, Crous PW (2016) Revising the *Schizoparmaceae*: *Coniella* and its synonyms *Pilidiella* and *Schizoparme*. Studies in Mycology 85: 1–34. 10.1016/j.simyco.2016.09.001 PMC5066162 27766001

[B2] Baroncelli R, Amby DB, Zapparata A, Sarrocco S, Vannacci G, Le Floch G, Harrison RJ, Holub E, Sukno SA, Sreenivasaprasad S (2016) Gene family expansions and contractions are associated with host range in plant pathogens of the genus *Colletotrichum*. BMC Genomics 17: e555. 10.1186/s12864-016-2917-6 PMC4974774 27496087

[B3] Bruen TC, Philippe H, Bryant D (2006) A quick and robust statistical test to detect the presence of recombination. Genetics 172: 2665–2681. 10.1534/genetics.105.048975 PMC1456386 16489234

[B4] Cai L, Giraud T, Zhang N, Begerow D, Cai G, Shivas RG (2011) The evolution of species concepts and species recognition criteria in plant pathogenic fungi. Fungal Diversity 50: 121–133. 10.1007/s13225-011-0127-8

[B5] Carbone I, Kohn LM (1999) A method for designing primer sets for speciation studies in filamentous ascomycetes. Mycologia 91(3): 553–556. 10.1080/00275514.1999.12061051

[B6] Carstens BC, Pelletier TA, Reid NM, Satler JD (2013) How to fail at species delimitation. Molecular Ecology 22(17): 4369–4383. 10.1111/mec.12413 23855767

[B7] Chen J, Hao X, Liu X, Ma L (2020) First report of *Pestalotiopsis neglecta* causing black spot needle blight of Pinus sylvestris var. mongolica in China. Plant Disease 104: e1545. 10.1094/PDIS-10-19-2249-PDN

[B8] Chen Y, Shao DD, Zhang AF, Yang X, Zhou MG, Xu YL (2014) First report of a fruit rot and twig blight on pomegranate (*Punica granatum*) caused by *Pilidiella granati* in Anhui Province of China. Plant Disease 98(5): e695. 10.1094/PDIS-09-13-1012-PDN 30708540

[B9] Chethana KWT, Zhou Y, Zhang W, Liu M, Xing QK, Li XH, Yan JY, Hyde KD (2017) *Coniella vitis* sp. nov. is the common pathogen of white rot in Chinese vineyards. Plant Disease 101(12): 2123–2136. 10.1094/PDIS-12-16-1741-RE 30677388

[B10] Chun J (1995) Computer-Assisted Classification and Identification of Actinomycetes. Ph.D. Thesis, University of Newcastle, Newcastle upon Tyne, UK. http://theses.ncl.ac.uk/jspui/handle/10443/410 [Accessed on 1 May 2026]

[B11] Crous PW, Giraldo A, Hawksworth DL, Robert V, Kirk PM, Guarro J, Robbertse B, Schoch CL, Damm U, Trakunyingcharoen T, Groenewald JZ (2014) The genera of fungi: Fixing the application of type species of generic names. IMA Fungus 5(1): 141–160. 10.5598/imafungus.2014.05.01.14 PMC4107892 25083414

[B12] Damien M de V, Refrégier G, López-Villavicencio M, Tellier A, Hood ME, Giraud T (2013) Cospeciation vs host-shift speciation: methods for testing, evidence from natural associations and relation to coevolution. New Phytologist 198(2): 347–385. 10.1111/nph.12150 23437795

[B13] Damm U, Cannon PF, Woudenberg JHC, Johnston PR, Weir BS, Tan YP, Shivas RG, Crous PW (2012) The *Colletotrichum boninense* species complex. Studies in Mycology 73: 1–36. 10.3114/sim0002 PMC3458415 23136457

[B14] Dissanayake AJ, Zhu JT, Chen YY, Maharachchikumbura SSN, Hyde KD, Liu JK (2024) A re-evaluation of *Diaporthe*: Refining the boundaries of species and species complexes. Fungal Diversity 126: 1–125. 10.1007/s13225-024-00538-7

[B15] Dong S, Raffaele S, Kamoun S (2015) The two-speed genomes of filamentous pathogens: Waltz with plants. Current Opinion in Genetics & Development 35: 57–65. 10.1016/j.gde.2015.09.001 26451981

[B16] Druzhinina IS, Komoń-Zelazowska M, Ismaiel A, Jaklitsch W, Mullaw T, Samuels GJ, Kubicek CP (2012) Molecular phylogeny and species delimitation in the section Longibrachiatum of *Trichoderma*. Fungal Genetics and Biology 49(5): 358–368. 10.1016/j.fgb.2012.02.004 PMC3350856 22405896

[B17] George D, Mallery P (2019) IBM SPSS Statistics 26 step by step: A simple guide and reference (16^th^ ed.). Routledge, Abingdon. 10.4324/9780429056765

[B18] Glass NL, Donaldson GC (1995) Development of primer sets designed for use with the PCR to amplify conserved genes from filamentous ascomycetes. Applied and Environmental Microbiology 61(4): 1323–1330. 10.1128/aem.61.4.1323-1330.1995 PMC167388 7747954

[B19] Guarnaccia V, Groenewald JZ, Polizzi G, Crous PW (2017) High species diversity in *Colletotrichum* associated with citrus diseases in Europe. Persoonia 39: 32–50. 10.3767/persoonia.2017.39.02 PMC5832956 29503469

[B20] Hassan O, Kim JS, Romain BBND, Chang T (2022) An account of *Colletotrichum* species associated with anthracnose of *Atractylodes ovata* in South Korea based on morphology and molecular data. PLOS ONE 17(1): e0263084. 10.1371/journal.pone.0263084 PMC8789177 35077506

[B21] Huson DH, Bryant D (2006) Application of phylogenetic networks in evolutionary studies. Molecular Biology and Evolution 23(2): 254–267. 10.1093/molbev/msj030 16221896

[B22] Hyde KD, Cai L, Cannon PF, Crouch JA, Crous PW, Damm U, Goodwin PH, Chen H, Johnston PR, Jones EBG, Liu ZY, McKenzie EHC, Moriwaki J, Noireung P, Pennycook SR, Pfenning LH, Prihastuti H, Sato T, Shivas RG, Tan YP, Taylor PWJ, Weir BS, Yang YL, Zhang JZ (2009) *Colletotrichum* – names in current use. Fungal Diversity 39: 147–182.

[B23] Hyde KD, Noorabadi MT, Thiyagaraja V, He MQ, Johnston PR, Wijesinghe SN, Armand A, Biketova AY, Chethana KWT, Erdoğdu M, Ge ZW, Groenewald JZ, Hongsanan S, Kušan I, Leontyev DV, Li DW, Lin CG, Liu NG, Maharachchikumbura SSN, Matočec N, et al. (2024) The 2024 outline of fungi and fungus-like taxa. Mycosphere 15(1): 5146–6239. 10.5943/mycosphere/15/1/25

[B24] Jankowiak R, Stępniewska H, Bilański P (2022) *Fungi* as potential factors limiting natural regeneration of pedunculate oak (*Quercus robur*) in mixed-species forest stands in Poland. Plant Pathology 71(3): 627–640. 10.1111/ppa.13529

[B25] Jiang N, Fan X, Tian C (2021) Identification and characterization of leaf-inhabiting fungi from *Castanea* plantations in China. Journal of Fungi 7(1): 1–64. 10.3390/jof7010064 PMC7831338 33477575

[B26] Levene H (1960) Robust tests for equality of variances. In: Olkin I (Ed.) Contributions to Probability and Statistics. Stanford University Press, Palo Alto, 278–292.

[B27] Li D, Dong Z, Liu Q, Wang Y, Zhang Z, Zhang X, Xia J (2025) Morpho-phylogenetic evidence reveals four novel species of *Coniella* (*Diaporthales*, *Schizoparmaceae*) from southern China. MycoKeys 116: 1–23. 10.3897/mycokeys.116.145857 PMC11992536 40224620

[B28] Li FY, Chi SM, Zhang HB (2021) Investigation of in vitro antioxidant activity of dihydromyricetin and flavonoids rich extract from vine tea (*Ampelopsis grossedentata*). Traditional Medicine Research 6(1): 1–7. 10.53388/tmr20200814193

[B29] Li H, Peng BY, Xie JY, Bai YQ, Li DW, Zhu LH (2024) *Pestalotiopsis jiangsuensis* sp. nov. causing needle blight on *Pinus massoniana* in China. Journal of Fungi 10(3): e230. 10.3390/jof10030230 PMC10970983 38535238

[B30] Li Y, Lin L, Cao J, Gan M, Fan X (2024) Three new species of *Colletotrichum* (*Glomerellales*, *Glomerellaceae*) associated with walnut (*Juglans regia*) anthracnose from China. MycoKeys 108: 147–167. 10.3897/mycokeys.108.125382 PMC11387834 39262404

[B31] Liu F, Hou L, Raza M, Cai L (2017) *Pestalotiopsis* and allied genera from *Camellia*, with description of 11 new species from China. Scientific Reports 7: e866. 10.1038/s41598-017-00972-5 PMC5429834 28408743

[B32] Liu F, Ma ZY, Hou LW, Diao YZ, Wu WP, Damm U, Song S, Cai L (2022) Updating species diversity of *Colletotrichum*, with a phylogenomic overview. Studies in Mycology 101: 1–56. 10.3114/sim.2022.101.01 PMC9365046 36059896

[B33] Liu YJ, Zhou ZH, Wang YC, Liu S, Deng WC, Hu WJ, Liu YZ, Yi TY (2024) First report of *Diaporthe eres* causing leaf blight on vine tea in China. *New Disease Reports* 49: e12275. 10.1002/ndr2.12275

[B34] Lücking R, Aime MC, Robbertse B, Miller AN, Ariyawansa HA, Aoki T, Cardinali G, Crous PW, Druzhinina IS, Geiser DM, Hawksworth DL, Hyde KD, Irinyi L, Jeewon R, Johnston PR, Kirk PM, Malosso E, May TW, Meyer W, Öpik M, Robert V, Stadler M, Thines M, Vu D, Yurkov AM, Zhang N, Schoch CL (2020) Unambiguous identification of fungi: Where do we stand and how accurate and precise is fungal DNA barcoding? IMA Fungus 11(1): 1–14. 10.1186/s43008-020-00033-z PMC7353689 32714773

[B35] Mahadevakumar S, Deepika YS, Amruthesh KN, Lakshmidevi N (2022) First report of *Coniella granati* associated with dieback of rose (*Rosa* sp.) in India. Plant Disease 106: e1304. 10.1094/PDIS-08-21-1816-PDN 34597151

[B36] Maharachchikumbura SSN, Guo LD, Chukeatirote E, Bahkali AH, Hyde KD (2011) *Pestalotiopsis* – morphology, phylogeny, biochemistry and diversity. Fungal Diversity 50: 167–187. 10.1007/s13225-011-0125-x

[B37] Maharachchikumbura SSN, Hyde KD, Groenewald JZ, Xu J, Crous PW (2014) *Pestalotiopsis revisited*. Studies in Mycology 79: 121–186. 10.1016/j.simyco.2014.09.005 PMC4255583 25492988

[B38] Maharachchikumbura SSN, Chen Y, Ariyawansa HA, Hyde KD, Haelewaters D, Perera RH, Samarakoon MC, Wanasinghe DN, Bustamante DE, Liu J-K, Lawrence DP, Cheewangkoon R, Stadler M (2021) Integrative approaches for species delimitation in *Ascomycota*. Fungal Diversity 109: 155–179. 10.1007/s13225-021-00486-6

[B39] Norphanphoun C, Zou M-T, Liu F-Q, Wang Y (2025) *Colletotrichum macroconidii* sp. nov. and six new records of *Colletotrichum* (*Glomerellaceae*, *Glomerellales*) from southwestern China. MycoKeys 122: 321–373. 10.3897/mycokeys.122.161122 PMC12464559 41018911

[B40] Page AJ, Taylor B, Delaney AJ, Soares J, Seemann T, Keane JA, Harris SR (2016) SNP-sites: Rapid efficient extraction of SNPs from multi-FASTA alignments. Microbial Genomics 2(4): e000056. 10.1099/mgen.0.000056 PMC5320690 28348851

[B41] Pereira DS, Phillips AJL (2024) *Diaporthe* species on palms – integrative taxonomic approach for species boundaries delimitation in the genus *Diaporthe*, with the description of *D. pygmaeae* sp. nov. Studies in Mycology 109: 487–594. 10.3114/sim.2024.109.08 PMC11663421 39717652

[B42] Porebski S, Bailey LG, Baum BR (1997) Modification of a CTAB DNA extraction protocol for plants containing high polysaccharide and polyphenol components. Plant Molecular Biology Reporter 15: 8–15. 10.1007/BF02772108

[B43] Prihastuti H, Cai L, Chen H, McKenzie EHC, Hyde KD (2009) Characterization of *Colletotrichum* species associated with coffee berries in northern Thailand. Fungal Diversity 39: 89–109

[B44] Puillandre N, Brouillet S, Achaz G (2012) ABGD, automatic barcode gap discovery for primary species delimitation. Molecular Ecology 21: 1864–1877. 10.1111/j.1365-294X.2011.05239.x 21883587

[B45] Puillandre N, Brouillet S, Achaz G (2021) ASAP: Assemble Species by Automatic Partitioning. Molecular Ecology Resources 21(2): 609–620. 10.1111/1755-0998.13281 33058550

[B46] Quaedvlieg W, Binder M, Groenewald JZ, Summerell BA, Carnegie AJ, Burgess TI, Crous PW (2014) Introducing the consolidated species concept to resolve species in the *Teratosphaeriaceae*. Persoonia 33: 1–40. 10.3767/003158514X681981 PMC4312929 25737591

[B47] R Core Team (2024) R: A language and environment for statistical computing. R Foundation for Statistical Computing, Vienna, Austria. [Accessed 17 April 2026] 10.32614/r.manuals

[B48] Rambaut A (2009) FigTree v1.3.1. http://tree.bio.ed.ac.uk/software/figtree/ [software]The FigTree website records v1.3.1 under 2009-12-21

[B49] Rannala B, Yang Z (1996) Probability distribution of molecular evolutionary trees: A new method of phylogenetic inference. Journal of Molecular Evolution 43: 304–311. 10.1007/BF02338839 8703097

[B50] Rehner SA, Samuels GJ (1994) Taxonomy and phylogeny of Gliocladium analysed from nuclear large subunit ribosomal DNA sequences. Mycological Research 98(6): 625–634. 10.1016/S0953-7562(09)80409-7

[B51] Shapiro SS, Wilk MB (1965) An analysis of variance test for normality (complete samples). Biometrika 52: 591–611. 10.1093/biomet/52.3-4.591

[B52] Shi X, Long X, Peng D, Lv J, Ye S, Yang Z, Ding Z (2025) First report of *Colletotrichum nymphaeae* causing anthracnose on *Ampelopsis grossedentata* in China. Plant Disease 109(9). 10.1094/PDIS-01-25-0001-PDN

[B53] Silva DN, Talhinhas P, Várzea V, Cai L, Paulo OS, Batista D (2012) Application of the *ApMat* marker to *Colletotrichum* species associated with coffee berry disease in Africa. Phytopathology 102(12): 1190–1198. 10.1094/PHYTO-03-12-0056-R

[B54] Sung GH, Hywel Jones NL, Sung JM, Luangsa Ard JJ, Shrestha B, Spatafora JW (2007) Phylogenetic classification of *Cordyceps* and the clavicipitaceous fungi. Studies in Mycology 57: 5–59. 10.3114/sim.2007.57.01 PMC2104736 18490993

[B55] Talhinhas P, Baroncelli R (2021) *Colletotrichum* species and complexes: Geographic distribution, host range and conservation status. Fungal Diversity 110(1): 109–198. 10.1007/s13225-021-00491-9

[B56] Talhinhas P, Baroncelli R (2023) Hosts of *Colletotrichum*. Mycosphere 14(2): 158–261. 10.5943/mycosphere/14/si2/4

[B57] Tamura K, Stecher G, Kumar S (2021) MEGA11: Molecular Evolutionary Genetics Analysis version 11. Molecular Biology and Evolution 38: 3022–3027. 10.1093/molbev/msab120 PMC8233496 33892491

[B58] Taylor JW, Jacobson DJ, Kroken S, Kasuga T, Geiser DM, Hibbett DS, Fisher MC (2000) Phylogenetic species recognition and species concepts in fungi. Fungal Genetics and Biology 31: 21–32. 10.1006/fgbi.2000.1228 11118132

[B59] Tekpinar A, Kalmer A (2022) Improving taxonomic delimitation of fungal species in the age of genomics and phenomics. Frontiers in Microbiology 13: e847067. 10.3389/fmicb.2022.847067 PMC8892103 35250961

[B60] Thines M (2019) An evolutionary framework for host shifts—jumping ships for survival. New Phytologist 224(2): 605–617. 10.1111/nph.16092 31381166

[B61] Usman HM, Hussain MD, Karim MM, Nizamani MM, Mubeen M, Hussain S, Kamran A, Wang Y, Liu FQ (2026) *Colletotrichum*: A versatile fungal genus with diverse infection strategies, host interactions, and management challenges. Phytopathology Research 8: 1–6. 10.1186/s42483-025-00391-9

[B62] Vences M, Miralles A, Brouillet S, Ducasse J, Fedosov A, Kharchev V, Kumari S, Patmanidis S, Puillandre N, Scherz MD, Kostadinov I, Renner SS (2021) iTaxoTools 0.1: Kickstarting a specimen-based software toolkit for taxonomists. Megataxa 6: 77–92. 10.11646/megataxa.6.2.1

[B63] Vieira WA, Michereff SJ, de Morais MA, Hidalgo-Morote DA, Doyle VP, Câmara MP (2020) Endophytic species of *Colletotrichum* associated with mango in northeastern Brazil. *Mycological Progress* 19(2): 141–156. 10.1007/s13225-014-0293-6

[B64] Vilgalys R, Hester M (1990) Rapid genetic identification and mapping of enzymatically amplified ribosomal DNA from several *Cryptococcus* species. Journal of Bacteriology 172(8): 4238–4246. 10.1128/jb.172.8.4238-4246.1990 PMC213247 2376561

[B65] Wallhead MW, Eldridge L, Ellis MA, Cannon PF, Grasdale W (2014) *Colletotrichum noveboracense* sp. nov., a pathogen causing bitter rot of apple in eastern North America. Mycotaxon 129(1): 181–194.

[B66] Wang S, Mu TC, Liu RY, Liu SB, Zhang ZX, Xia JW, Li Z, Zhang XG (2022) *Coniella castanea* sp. nov. on *Castanea mollissima* from Shandong Province, China. Phytotaxa 559(1): 025–034. 10.11646/phytotaxa.559.1.3

[B67] Weir BS, Johnston PR, Damm U (2012) The *Colletotrichum gloeosporioides* species complex. Studies in Mycology 73: 115–180. 10.3114/sim0011 PMC3458417 23136459

[B68] Wen J, Boggan J, Nie ZL (2014) Synopsis of *Nekemias* Raf., a segregate genus from *Ampelopsis* Michx. (*Vitaceae*) disjunct between eastern/southeastern Asia and eastern North America, with ten new combinations. PhytoKeys 42: 11–19. 10.3897/phytokeys.42.7704 PMC4223361 25383008

[B69] White TJ, Bruns T, Lee S, Taylor J (1990) Amplification and direct sequencing of fungal ribosomal RNA genes for phylogenetics. In: Innis MA, Gelfand DH, Sninsky JJ, White TJ (Eds) PCR protocols: A guide to methods and applications. Academic Press, New York, 315–322. 10.1016/B978-0-12-372180-8.50042-1

[B70] Wickham H (2016) ggplot2: Elegant graphics for data analysis. Springer, New York. 10.1007/978-3-319-24277-4

[B71] Wu RR, Li X, Cao YH, Peng X, Liu GF, Liu ZK, Yang Z, Liu ZY, Wu Y (2023) China medicinal plants of *Ampelopsis grossedentata*—A review of their botanical characteristics, use, phytochemistry, active pharmacological components, and toxicology. Molecules 28(20): e7145. 10.3390/molecules28207145 PMC10609530 37894624

[B72] Xi Y, Yang Y, Zhang Q, Zhang Z, Ha Z (2025) Research on the influence of ecological specialty industry vine tea on farmers’ livelihoods in Laifeng County, China. Frontiers in Sustainable Food Systems 9: e1465868. 10.3389/fsufs.2025.1465868

[B73] Yi T, Li S, Hong Y (2019) Moyeam canker caused by *Phomopsis viticola*. Journal of Plant Diseases and Protection 126(3): 261–262. 10.1007/s41348-019-00215-x

[B74] Yuan SQ, Wang YC, Lei L, Hong JY, Yi TY, Hong YY (2022) First report of *Pestalotiopsis microspora* causing leaf spot on Moyeam in China. Plant Disease 106(7): e1996. 10.1094/PDIS-04-21-0859-PDN

[B75] Zeng X-Y, Tan T-J, Tian F-H, Wang Y, Wen T-C (2023) OFPT: A one-stop software for fungal phylogeny. Mycosphere 14: 1730–1741. 10.5943/mycosphere/14/1/20

[B76] Zhang Q, Hyde KD, Sun YR, Zeng SX, Long H, Bhunjun CS, Al-Otibi F, Li C, Wang Y, Liu FQ, Maharachchikumbura SSN (2026) Genome-scale phylogenetics and integrative species delimitation to clarify taxonomic boundaries in pestalotiopsis-like fungi. Fungal Diversity 136: e136003. 10.65390/fdiv.2026.136003

[B77] Zhang YM, Maharachchikumbura SSN, Wei JG, McKenzie EHC, Hyde KD (2012) *Pestalotiopsis camelliae*, a new species associated with grey blight of *Camellia japonica* in China. Sydowia 64(2): 335–344

[B78] Zhang YM, Maharachchikumbura SSN, Tian XG, Hyde KD (2013) *Pestalotiopsis* species on palms. Fungal Diversity 60: 137–157. 10.1007/s13225-013-0226-2

[B79] Zhao L, Zou Q, Guan L, Xie Y (2025) First report of *Pestalotiopsis oryzae* causing leaf spot on *Lanxangia tsaoko* in Yunnan, China. Journal of Plant Pathology 107: e1565. 10.1007/s42161-025-01917-y

