# Supplementary material for: Integrative taxonomy reveals three novel pathogens belonging to Coniella, Colletotrichum and Pestalotiopsis on Nekemias grossedentata in China
Source: MycoKeys. 2026 Sep 11;140:49–77. doi: 10.3897/mycokeys.140.200783 (PMC13589297; doi:10.3897/mycokeys.140.200783)
Supplement: Supplementary file 1 [file mycokeys-140-049-s001.pdf]

Table S1: Strains used in this study and their GenBank accession numbers.

**Species**	**Isolate**	**Host**	**Location**	**GenBank accession number**							
				**ITS**	***RPB2***	***TEF1***	***TUB2***	***CAL***	***CHS***	***ACT***	**LSU**
**Ascomycota > Sordariomycetes > Diaporthales > Schizoparmaceae > *Coniella***											
Coniella africana	CBS114133T	*Eucalyptus nitens*	South Africa	AY339344	KX833421	KX833600	-	-	-	-	AY339293
Coniella petrakii	CBS112338	*Vitis vinifera*	France	KX833515	KX833427	KX833607	-	-	-	-	KX833339
Coniella petrakii	CBS112346	*Vitis vinifera*	France	KX833516	KX833428	KX833608	-	-	-	-	KX833340
Coniella petrakii	CBS112362	*Vitis vinifera*	France	KX833517	KX833429	KX833609	-	-	-	-	KX833341
Coniella petrakii	CBS116312	*Vitis vinifera*	France	KX833528	KX833438	KX833621	-	-	-	-	KX833353
Coniella petrakii	CBS166.84	*Vitis vinifera*	France	AY339331	-	KX833623	-	-	-	-	AY339286
Coniella diplodiopsis	CBS109.23	*Vitis vinifera*	Switzerland	AY339332	KX833440	KX833624	-	-	-	-	AY339287
Coniella diplodiopsis	CBS112637	*Vitis vinifera*	South Africa	KX833530	KX833441	KX833625	-	-	-	-	KX833355
Coniella erumpens	CBS523.78 T	Rotten wood	Chile	KX833535	KX833446	KX833630	-	-	-	-	KX833361
Coniella eucalyptigena	CBS139893T	*Eucalyptus brassiana*	Malaysia	KR476725	-	-	-	-	-	-	KR476760
Coniella eucalyptorum	CBS110674	*Eucalyptus* sp.	Brazil	KX833536	KX833447	KX833631	-	-	-	-	KX833362
Coniella eucalyptorum	CBS111023	*Eucalyptus phylla*	Mexico	KX833537	KX833448	KX833632	-	-	-	-	KX833363
Coniella eucalyptorum	CBS111202	Unkonw	Indonesia	KX833539	KX833449	KX833634	-	-	-	-	KX833365
Coniella fragariae	CBS164.37	*Ulmus campestris*	Italy	KX833565	KX833471	KX833661	-	-	-	-	KX833388
Coniella fragariae	CBS183.52	*Tamarix* sp.	Unkonw	KX833567	KX833473	KX833665	-	-	-	-	KJ710442
Coniella fusiformis	CBS114850	Eucalyptus pellita	Australia	KX833574	KX833479	KX833672	-	-	-	-	KX833395
Coniella fusiformis	CBS141596 T	*Eucalyptus* sp.	Indonesia	KX833576	KX833481	KX833674	-	-	-	-	KX833397
Coniella granati	CBS130974	*Punica granatum*	Iran	JN815312	KX833482	KX833675	-	-	-	-	KX833398
Coniella granati	CBS130975	*Punica granatum*	Iran	JN815313	KX833483	KX833676	-	-	-	-	KX833399
Coniella granati	CBS152.33	*Punica granatum*	Cyprus	KX833578	KX833485	KX833678	-	-	-	-	AF408379
Coniella javanica	CBS455.68 T	*Hibiscus sabdariffai*	Indonesia	KX833583	KX833489	KX833683	-	-	-	-	KX833403
Coniella koreana	CBS143.97 T	Unkonw	South Korea	KX833584	KX833490	KX833684	-	-	-	-	AF408378

Coniella lanneae	CBS141597 T	*Lannea* sp.	Zambia	KX833585	KX833491	KX833685	–	–	–	–	KX833404
Coniella limoniformis	CBS111021 T	*Fragaria* sp.	South Africa	KX833586	KX833492	KX833686	–	–	–	–	KX833405
Coniella macrospora	CBS524.73 T	*Terminalia ivoriensisstem*	Ivory Coast	KX833587	KX833493	KX833687	–	–	–	–	AY339292
Coniella malaysiana	CBS141598 T	*Corymbia torelliana*	Malaysia	KX833588	KX833494	KX833688	–	–	–	–	KX833406
Coniella nicotianae	CBS875.72 T	*Nicotiana tabacum*	Jamaica	KX833590	KX833495	KX833690	–	–	–	–	KX833407
Coniella nigra	CBS165.60 T	Soil	India	AY339319	KX833496	KX833691	–	–	–	–	KX833408
Coniella obovata	CBS111025	Leaf litter	South Africa	AY339313	KX833497	KX833692	–	–	–	–	KX833409
Coniella paracastaneicola	CBS141292 T	*Eucalyptus* sp.	Australia	KX833591	KX833498	KX833693	–	–	–	–	KX833410
Coniella peruensis	CBS110394 T	Soil of rain forest	Peru	KJ710463	KX833499	KX833695	–	–	–	–	KJ710441
Coniella pseudostraminea	CBS112624 T	*Fragaria* sp.	South Africa	KX833593	KX833500	KX833696	–	–	–	–	KX833412
Coniella quercicola	CBS283.76	Excrements of *Glomerus*	Netherlands	KX833594	KX833501	KX833697	–	–	–	–	KX833413
Coniella quercicola	CBS904.69 NT	*Quercus robur*	Netherlands	KX833595	KX833502	KX833698	–	–	–	–	KX833414
Coniella solicola	CBS114007	Unkonw	USA	AY339320	KX833504	KX833700	–	–	–	–	KX833415
Coniella solicola	CBS766.71 T	Soil	South Africa	KX833597	KX833505	KX833701	–	–	–	–	KX833416
Coniella straminea	CBS149.22	*Fragaria* sp.	USA	AY339348	KX833506	KX833704	–	–	–	–	AY339296
Coniella tibouchinae	CBS131594 T	*Tibouchina granulosa*	Brazil	JQ281774	KX833507	JQ281778	–	–	–	–	KX833418
Coniella tibouchinae	CBS131595 T	*Tibouchina granulosa*	Brazil	JQ281775	KX833508	JQ281779	–	–	–	–	KX833419
Coniella wangiensis	CBS132530 T	*Eucalyptus* sp.	Australia	JX069873	KX833509	KX833705	–	–	–	–	JX069857
Coniella castaneicola	CFCC54344T	*Castanea mollissima*	China	MW208111	–	MW227343	–	–	–	–	–
Coniella castaneicola	ZY7-2	*Castanea mollissima*	China	MW208112	–	MW227344	–	–	–	–	–
Coniella castanea	SAUCC 200313T	*Castanea mollissima*	China	OL757537	OL770463	OL780610	–	–	–	–	OL757563
Coniella castanea	SAUCC 200314	*Castanea mollissima*	China	OL757538	OL770464	OL780611	–	–	–	–	OL757564
Coniella grossedentatae	SAUCC 1354-1	*Nekemias grossedentata*	China	OQ357062	PQ361000	PQ404774	–	–	–	–	PQ357102
Coniella grossedentatae	CGMCC3.27783 T	*Nekemias grossedentata*	China	OQ357063	PQ361001	PQ404775	–	–	–	–	PQ357103
Coniella ampelopsidis	**YzU 251452 T**	***Nekemias grossedentata***	**China**	**PZ342989**	**PZ388304**	**PZ388300**	–	–	–	–	**PZ368391**
Coniella ampelopsidis	**YzU 251453**	***Nekemias grossedentata***	**China**	**PZ342990**	**PZ388305**	**PZ388301**	–	–	–	–	**PZ368392**

Coniella ampelopsidis	**YzU 251454**	***Nekemias grossedentata***	**China**	**PZ342991**	**PZ388306**	**PZ388302**	–	–	–	–	**PZ368393**
Coniella ampelopsidis	**YzU 251455**	***Nekemias grossedentata***	**China**	**PZ342992**	**PZ388307**	**PZ388303**	–	–	–	–	**PZ368394**
Melanconiella hyperopta	CBS131696	*Carpinus betulus*	Austria	JQ926281	KX833510	–	–	–	–	–	JQ926281

Ascomycota > Sordariomycetes > Glomerellales > Glomerellaceae > *Colletotrichum*

Colletotrichum aenigma	ICMP 18608 T	*Persea americana*	Israel	JX010244	–	–	JX010389	JX009683	JX009774	JX009443	–
Colletotrichum aeschynomenes	ICMP 17673 T	*Aeschynomene virginica*	USA	JX010176	–	–	JX010392	JX009721	JX009799	JX009483	–
Colletotrichum alatae	CBS 304.67 T	*Dioscorea alata*	India	JX010190	–	–	JX010383	JX009738	JX009837	JX009471	–
Colletotrichum alienum	ICMP 12071 T	*Malus domestica*	New Zealand	JX010251	–	–	JX010411	JX009654	JX009882	JX009572	–
Colletotrichum anhuiense	AG 52	*Osmanthus fragrans*	China	OL772737	–	–	OL854186	OL854147	OL854158	OM100918	–
Colletotrichum anhuiense	AG 85 T	*Osmanthus fragrans*	China	OL772736	–	–	OL854185	OL854146	OL854157	OM100917	–
Colletotrichum aotearoa	ICMP 18537 T	*Coprosma* sp.	New Zealand	JX010205	–	–	JX010420	JX009611	JX009853	JX009564	–
Colletotrichum arecicola	CGMCC 3.19667 T	*Areca catechu*	China	MK914635	–	–	MK935498	–	MK935541	MK935374	–
Colletotrichum artocarpicola	MFLUCC 18-1167 T	*Artocarpus heterophyllus*	Thailand	MN415991	–	–	MN435567	–	MN435569	MN435570	–
Colletotrichum asianum	CBS 130418 T	*Coffea arabica*	Thailand	FJ972612	–	–	JX010406	FJ917506	JX009867	JX009584	–
Colletotrichum australianum	UMC 002 T	*Citrus sinensis*	Australia	MG572138	–	–	MG572149	–	MW091987	MN442109	–
Colletotrichum avicenniae	MFLUCC23-0289	*Avicennia marina*	Thailand	OR856121	–	–	OR886396	–	OR886402	OR886393	–
Colletotrichum camelliae	CNUCC 257-2-4	*Camellia sinensis*	China	PP843285	–	–	PP890425	PP854762	PP841693	PP922865	–
Colletotrichum camelliae	CNUCC 781B-1-2	*Camellia sinensis*	China	PP843307	–	–	PP870977	PP854783	PP841715	PP922887	–
Colletotrichum cangyuanense	CNUCC 59B-51-4	*Ageratina adenophora*	China	PP843292	–	–	PP890430	PP854807	PP841700	PP922872	–
Colletotrichum cangyuanense	CNUCC 96-7-1	*Ageratina adenophora*	China	PP843260	–	–	PP870997	PP854803	PP841735	PP922907	–
Colletotrichum changpingense	CGMCC 3.17582	Fragaria × ananass	China	KP683152	–	–	KP852490	–	KP852449	KP683093	–
Colletotrichum chrysophilum	CMM 4268 T	*Musa* sp.	Brazil	KX094252	–	–	KX094285	KX094063	KX094083	KX093982	–
Colletotrichum ciggaro	ICMP 18539 T	*Olea europaea*	Australia	JX010230	–	–	JX010434	JX009635	JX009800	JX009523	–
Colletotrichum clidemiae	ICMP 18658 T	*Clidemia hirta*	USA	JX010265	–	–	JX010438	JX009645	JX009877	JX009537	–
Colletotrichum cobbittiense	BRIP 66219 T	*Cordyline stricta*	Australia	MH087016	–	–	MH094137	–	MH094135	MH094134	–

Colletotrichum conoides	CGMCC 3.17615 T	*Chili pepper*	China	KP890168	-	-	KP890174	KP890150	KP890156	KP890144	-
Colletotrichum cordea	CFCC 70160	*Juglans regia*	China	PP397135	-	-	PP425127	-	PP425045	PP425002	-
Colletotrichum cordea	CFCC 59618 T	*Juglans regia*	China	PP397136	-	-	PP425128	-	PP425046	PP425003	-
Colletotrichum cordylinicola	ICMP 18579 T	*Cordyline fruticosa*	Thailand	JX010226	-	-	JX010440	HM470238	JX009864	HM470235	-
Colletotrichum dongguanense	CFCC 72438 T	*Bauhinia purpurea*	China	PV524715	-	-	PV573597	PV573627	PV573536	PV573565	-
Colletotrichum dongguanense	CFCC 72439	*Bauhinia purpurea*	China	PV524716	-	-	PV573598	PV573628	PV573537	PV573566	-
Colletotrichum endophyticum	LC 0324 T	*Pennisetum purpureum*	Thailand	KC633854	-	-	MZ673954	KC810018	MZ799261	KF306258	-
Colletotrichum flavosporum	CFCC 72442 T	*Bougainvillea glabra*	China	PV524713	-	-	PV573595	PV573625	PV573534	PV573563	-
Colletotrichum flavosporum	CFCC 72443	*Bougainvillea glabra*	China	PV524714	-	-	PV573596	PV573626	PV573535	PV573564	-
Colletotrichum fructicola	CBS 130416 T	*Coffea arabica*	Thailand	JX010165	-	-	JX010405	FJ917508	JX009866	FJ907426	-
Colletotrichum fructicola	CBS 125397	*Tetragastris panamensis*	Panama	JX010173	-	-	JX010409	JX009674	JX009874	JX009581	-
Colletotrichum fructivorum	CBS 133125 T	*Vaccinium macrocarpon*	Burlington	JX145145	-	-	JX145196	-	MZ799259	MZ664126	-
Colletotrichum gardeniae	GUCC 12048	*Gardenia jasminoides*	China	OP722989	-	-	OP720857	-	OP715765	OP715800	-
Colletotrichum gardeniae	GUCC 12049 T	*Gardenia jasminoides*	China	OP722995	-	-	OP720858	-	OP715766	OP715801	-
Colletotrichum gloeosporioides	ICMP 12938	*Citrus sinensis*	New Zealand	JX010147	-	-	-	JX009732	JX009746	JX009560	-
Colletotrichum gloeosporioides	CBS 273.51 T	*Citrus limon*	Italy	JX010148	-	-	-	JX009745	JX009903	JX009558	-
Colletotrichum grevilleae	CBS 132879T	*Grevillea* sp.	Italy	KC297078	-	-	KC297102	KC296963	KC296987	KC296941	-
Colletotrichum grossum	CGMCC 3.17614T	*Chili pepper*	China	KP890165	-	-	KP890171	KP890147	KP890153	KP890141	-
Colletotrichum guangyuanense	CFCC 59902 T	*Juglans regia*	China	PP397133	-	-	PP425125	-	PP425043	PP425000	-
Colletotrichum guangyuanense	CFCC 70249	*Juglans regia*	China	PP397134	-	-	PP425126	-	PP425044	PP425001	-
Colletotrichum guiyangense	CNUCC 823-1-1-1 T	*Camellia sinensis*	China	PP812207	-	-	PP830770	PP830766	PP819547	PP830762	-
Colletotrichum guiyangense	CNUCC 823-1-1-2	*Camellia sinensis*	China	PP812208	-	-	PP830771	PP830767	PP819548	PP830763	-
Colletotrichum hebeiense	MFLUCC 130726 T	*Vitis vinifera*	China	KF156863	-	-	KF288975	-	KF289008	KF377532	-
Colletotrichum helleniense	CBS 142418 T	*Poncirus trifoliata*	Greece	KY856446	-	-	KY856528	KY856099	KY856186	KY856019	-
Colletotrichum henanense	LC 3030 T	*Camellia sinensis*	China	KJ955109	-	-	KJ955257	KJ954662	MZ799256	KM023257	-
Colletotrichum horii	ICMP 10492 T	*Diospyros kaki*	Japan	GQ329690	-	-	JX010450	JX009604	JX009752	JX009438	-

Colletotrichum hystricis	CBS 142411 T	*Citrus hystrix*	Italy	KY856450	–	–	KY856532	KY856103	KY856190	KY856023	–
Colletotrichum jiangxiense	CGMCC 3.17361	*Camellia sinensis*	China	KJ955149	–	–	OK236389	KJ954701	MZ799257	KJ954427	–
Colletotrichum jinpingense	CCTCC AF2021056 T	*Camellia sinensis*	China	MZ165534	–	–	MZ277688	–	MZ352021	MZ277632	–
Colletotrichum jixiense	CNUCC 5-21-2-1 T	*Cunninghamia lanceolata*	China	PP812209	–	–	PP830772	PP830768	PP819549	PP830764	–
Colletotrichum kahawae	ICMP 17816 T	*Coffea arabica*	Kenya	JX010231	–	–	JX010444	JX009642	JX009813	JX009452	–
Colletotrichum kunmingense	GUCC 12053 T	*Ophiopogon japonicus*	China	OP722975	–	–	OP720861	–	OP715769	OP715804	–
Colletotrichum ledongense	CGMCC 3.18888 T	*Hevea brasiliensis*	China	MG242009	–	–	MG242011	MG242013	MG242019	MG242015	–
Colletotrichum ligustri	GUCC 12111 T	*Ilex chinensis*	China	OP722988	–	–	OP720864	–	OP715773	OP740216	–
Colletotrichum lumnitzerae	MFLUCC 23-0291	mangroves	Thailand	OR856122	–	–	OR886397	–	OR886403	OR886394	–
Colletotrichum makassarense	CBS 143664 T	*Capsicum annuum*	Indonesia	MH728812	–	–	MH846563	–	MH805850	MH781480	–
Colletotrichum nanjingense	CFCC 58939	*Jasminum mesnyi*	China	OQ456154	–	–	OQ507146	–	OQ507140	OQ507150	–
Colletotrichum nanjingense	CFCC 58940 T	*Jasminum mesnyi*	China	OQ456155	–	–	OQ507147	–	OQ507141	OQ507149	–
Colletotrichum nullisetosum	YMF 1.06946 T	*Mangifera indica*	China	OK030872	–	–	OK513641	–	OK513571	OK513611	–
Colletotrichum perseae	CBS 141365 T	*Avocado*	Israel	KX620308	–	–	KX620341	KX620206	MZ799260	KX620145	–
Colletotrichum polypodialium	MFLUCC 22-0178	*Nephrolepis* sp.	Thailand	OP802361	–	–	OP801739	–	OP801702	OP801685	–
Colletotrichum proteae	CBS 132882 T	*Protea* sp.	South Africa	KC297079	–	–	KC297101	KC296960	KC296986	KC296940	–
Colletotrichum pseudotheobromicola	MFLUCC 181602 T	*Prunus avium*	China	MH817395	–	–	MH853684	–	MH853678	MH853681	–
Colletotrichum psidii	CBS 145.29	*Psidium* sp.	South Africa	JX010219	–	–	JX010443	JX009743	JX009901	JX009515	–
Colletotrichum queenslandicum	ICMP 1778	*Carica papaya*	Australia	JX010276	–	–	JX010414	JX009691	JX009899	JX009447	–
Colletotrichum rhexiae	CBS 133134	*Rhexia virginica*	USA	JX145128	–	–	JX145179	–	MZ799258	MZ664127	–
Colletotrichum siamense	CBS 130417 T	*Coffea arabica*	Thailand	JX010171	–	–	JX010404	–	JX009865	FJ907423	–
Colletotrichum siamense	CFCC 72430	*Camellia* sp.	China	PV524717	–	–	PV573599	PV573629	PV573538	PV573567	–
Colletotrichum siamense	CFCC72431	*Camellia* sp.	China	PV524718	–	–	PV573600	PV573630	PV573539	PV573568	–
Colletotrichum subhenanense	YMF 1.07324	*Ageratina adenophora*	China	OK030884	–	–	OK513648	–	OK513582	OK513619	–
Colletotrichum subhenanense	YMF 1.06865	*Ageratina adenophora*	China	OK030883	–	–	OK513647	–	OK513581	OK513618	–
Colletotrichum ti	ICMP 4832	*Cordyline* sp.	New Zealand	JX010269	–	–	JX010442	JX009649	JX009898	JX009520	–
Colletotrichum tropicale	CBS 124949	*Theobroma cacao*	Panama	JX010264	–	–	JX010407	JX009719	JX009870	JX009489	–

Colletotrichum wuxiense	CGMCC 3.17894 T	*Hippuris vulgaris*	China	KU251591	-	-	KU252200	KU251833	KU251939	KU251672	-
Colletotrichum xanthorrhoeae	CBS 127831 T	*Xanthorrhoea preissii*	Australia	JX010261	-	-	JX010448	JX009653	JX009823	JX009478	-
Colletotrichum xishuangbannaense	MFLUCC 190107 T	*Magnolia liliifera*	China	MW346469	-	-	-	-	MW660832	MW652294	-
Colletotrichum yunajiangensis	YMF 1.04996 T	*Ageratina adenophora*	China	OK030885	-	-	OK513649	-	OK513583	OK513620	-
Colletotrichum mengyinense	SAUCC0702 T	*Rosachinensis*	China	MW786742	-	-	MW888970	-	MW883686	MW883695	
Colletotrichum mengyinense	CFCC 59605	*Juglans regia*	China	PP397140	-	-	PP425132	-	PP425049	PP425007	
Colletotrichum noveboracense	AFK220	*Apple/McIntosh*	Ulster/NY	MN625451	-	-	MN622861	-	-	MN622839	
Colletotrichum noveboracense	AFK423	*Apple/McIntosh*	Ulster/NY	MN708219	-	-	MN701194	-	-	MN701181	
Colletotrichum nupharicola	CBS 470.96 T	*Nuphar lutea sub sp. polysepala*	USA	JX010187	-	-	JX010398	-	JX009835	JX009437	
Colletotrichum enshiense	**YzU 251441 T**	***Nekemias grossedentata***	**China**	PZ342982	-	-	**PZ378886**	**PZ378882**	**PZ378884**	**PZ378888**	-
Colletotrichum enshiense	**YzU 251442**	***Nekemias grossedentata***	**China**	**PZ342983**	-	-	**PZ378887**	**PZ378883**	**PZ378885**	**PZ378889**	-
Colletotrichum boninense	CBS 123755 T	*Crinum asiaticum var.*	Japan	JQ005153	-	-	JQ005588	-	JQ005327	JQ005501	-

Ascomycota > Sordariomycetes > Amphisphaeriales > Sporocadaceae > *Pestalotiopsis*

Pestalotiopsis abietis	CFCC 53012	*Abies fargesii*	China	MK397014	-	MK622278	MK622281	-	-	-	-
Pestalotiopsis abietis	GUCC 23-0397	*Camptotheca acuminata*	China	PQ129380	-	PQ141015	PQ148965	-	-	-	-
Pestalotiopsis abietis	GUCC 23-0398	*Camptotheca acuminata*	China	PQ129381	-	PQ141016	PQ148966	-	-	-	-
Pestalotiopsis abietis	CFCC 53011 T	*Abies fargesii*	China	MK397013	-	MK622277	MK622280	-	-	-	-
Pestalotiopsis americana	CBS 111576 T	*Leucospermum cunei*	America	MH553961	-	MH554379	MH554620	-	-	-	-
Pestalotiopsis anhuiensis	CFCC 54791 T	*Cyclobalanopsis glauca*	China	ON007028	-	ON005045	ON005056	-	-	-	-
Pestalotiopsis australasiae	CBS 114126 T	*Knightia sp.*	New Zealand	KM199297	-	KM199499	KM199409	-	-	-	-
Pestalotiopsis australasiae	CBS 114141	*Protea* 'Pink Ice'	Australia	KM199298	-	KM199501	KM199410	-	-	-	-

Pestalotiopsis biciliata	CBS 124463 T	*Platanus*	Slovakia	KM199308	–	KM199505	KM199399	–	–	–	–
Pestalotiopsis biciliata	CBS 236.38	*Paeonia* sp.	Italy	KM199309	–	KM199506	KM199401	–	–	–	–
Pestalotiopsis brachiata	LC2988 T	*Camellia* sp.	China	KX894933	–	KX895150	KX895265	–	–	–	–
Pestalotiopsis brachiata	LC8188	*Camellia* sp.	China	KY464142	–	KY464152	KY464162	–	–	–	–
Pestalotiopsis camelliae-japonicae	ZHKUCC 23-0826 T	*Camellia japonica*	China	OR258040	–	OR251480	OR251483	–	–	–	–
Pestalotiopsis camelliae-japonicae	ZHKUCC 23-0828	*Camellia japonica*	China	OR258042	–	OR251482	OR251485	–	–	–	–
Pestalotiopsis camelliae-oleiferae	CSUFTCC08 T	*Camellia oleifera*	China	OK493593	–	OK507963	OK562368	–	–	–	–
Pestalotiopsis camelliae-oleiferae	CSUFTCC09	*Camellia oleifera*	China	OK493594	–	OK507964	OK562369	–	–	–	–
Pestalotiopsis camelliae-oleiferae	GUCC 23-0359	*Rhododendron delavayi*	China	PX425033	–	PX432011	PX432160	–	–	–	–
Pestalotiopsis camelliae-oleiferae	GUCC 23-0358	*Lindera glauca*	China	PQ129366	–	PQ141001	PQ148951	–	–	–	–
Pestalotiopsis chaoyangensis	CFCC 55549 T	*Euonymus japonicus*	China	OQ344763	–	OQ410582	OQ410584	–	–	–	–
Pestalotiopsis chaoyangensis	CFCC 58805	*Euonymus japonicus*	China	OQ344764	–	OQ410583	OQ410585	–	–	–	–
Pestalotiopsis chiangmaiensis	MFLUCC 22-0127 T	Bamboo	Thailand	OP497990	–	OP753374	OP752137	–	–	–	–
Pestalotiopsis disseminata	CBS 118552	*Eucalyptus botryoides*	New Zealand	MH553986	–	MH554410	MH554652	–	–	–	–
Pestalotiopsis disseminata	CBS 143904	*Persea americana*	New Zealand	MH554152	–	MH554587	MH554825	–	–	–	–
Pestalotiopsis disseminata	CPC 29351	*Eucalyptus* sp.	New Zealand	MH554166	–	MH554601	MH554839	–	–	–	–
Pestalotiopsis doitungensis	MFLUCC 14-0090 T	*Dendrobium* sp.	Thailand	MK993573	–	MK975831	MK975836	–	–	–	–
Pestalotiopsis dracontomelonis	MFLUCC 10-0149 T	*Dracontomelon dao*	Thailand	KP781877	–	KP781880	–	–	–	–	–
Pestalotiopsis dracontomelonis	MFLUCC 22-0119	*Dipterocarpus alatus*	Thailand	OP905676	–	OQ127638	OQ127639	–	–	–	–
Pestalotiopsis etonensis	BRIP 66615 T	*Sporobolus jacquemontii*	Australia	MK966339	–	MK977635	MK977634	–	–	–	–
Pestalotiopsis exudata	CGMCC 3.23488 T	*Aucuba japonica*	China	OR247985	–	OR361460	OR381060	–	–	–	–
Pestalotiopsis exudata	LC15850	*Aucuba japonica*	China	OR247986	–	OR361461	OR381061	–	–	–	–
Pestalotiopsis ficicola	SAUCC230046 T	*Ficus microcarpa*	China	OQ691974	–	OQ718691	OQ718749	–	–	–	–
Pestalotiopsis ficicola	SAUCC230042	*Ficus microcarpa*	China	OQ691972	–	OQ718689	OQ718747	–	–	–	–
Pestalotiopsis ficicrescens	GUCC 21556 T	*Ficus tikoua*	China	MZ477311	–	PQ141039	MZ868301	–	–	–	–
Pestalotiopsis ficicrescens	GUCC 23-0209	*Morella rubra*	China	PX425037	–	PX432015	PX432164	–	–	–	–

Pestalotiopsis ficicrescens	CGMCC 3.23489 T	*Cinnamomum camphora*	China	OR247987	–	OR361462	OR381062	–	–	–	–
Pestalotiopsis ficicrescens	LC5089	*Cinnamomum camphora*	China	OR247998	–	OR361473	OR381073	–	–	–	–
Pestalotiopsis foliicola	CFCC 54440 T	*Castanopsis faberi*	China	ON007029	–	ON005046	ON005057	–	–	–	–
Pestalotiopsis foliicola	CFCC 57359	*Castanopsis faberi*	China	ON007030	–	ON005047	ON005058	–	–	–	–
Pestalotiopsis formosana	NTUCC 17-009 T	Dead grass	Taiwan, China	MH809381	–	MH809389	MH809385	–	–	–	–
Pestalotiopsis formosana	NTUCC 17-010	Dead grass	Taiwan, China	MH809382	–	MH809390	MH809386	–	–	–	–
Pestalotiopsis fusiformis	CGMCC 3.23495 T	*Rhododendron* sp.	China	OR247995	–	OR361470	OR381070	–	–	–	–
Pestalotiopsis fusiformis	LC15852	*Rhododendron* sp.	China	OR247996	–	OR361471	OR381071	–	–	–	–
Pestalotiopsis grevilleae	CBS 114127 T	*Grevillea* sp.	Australia	KM199300	–	KM199504	KM199407	–	–	–	–
Pestalotiopsis guangxiensis	CFCC 54308 T	*Quercus griffithii*	China	OK339737	–	OK358498	OK358513	–	–	–	–
Pestalotiopsis guangxiensis	CFCC 54300	*Quercus griffithii*	China	OK339738	–	OK358499	OK358514	–	–	–	–
Pestalotiopsis guiyangensis	CFCC 70626 T	*Eriobotrya japonica*	China	PP784740	–	PP842629	PP842617	–	–	–	–
Pestalotiopsis guiyangensis	CFCC 70630	*Rohdea japonica*	China	PP784741	–	PP842630	PP842618	–	–	–	–
Pestalotiopsis hispanica	CBS 115391 T	*Protea* 'Susara'	Spain	MH553981	–	MH554399	MH554640	–	–	–	–
Pestalotiopsis hispanica	KUNCC 22-12593	*Protea* 'Susara'	Spain	OP498000	–	OP753378	OP737882	–	–	–	–
Pestalotiopsis hispanica	KUNCC 22-12595	*Protea* 'Susara'	Spain	OP498001	–	OP753381	OP765910	–	–	–	–
Pestalotiopsis hispanica	GUCC 23-0315	*Peristrophe japonica*	China	PQ129356	–	PQ140991	PQ148941	–	–	–	–
Pestalotiopsis hispanica	GUCC 23-0320	*Peristrophe japonica*	China	PX425039	–	PX432017	PX432166	–	–	–	–
Pestalotiopsis hunanensis	CSUFTCC15 T	*Camellia oleifera*	China	OK493599	–	OK507969	OK562374	–	–	–	–
Pestalotiopsis hunanensis	CSUFTCC18	*Camellia oleifera*	China	OK493600	–	OK507970	OK562375	–	–	–	–
Pestalotiopsis hunanensis	CFCC 59538 T	*Pinus massoniana*	China	OR533577	–	OR539186	OR539191	–	–	–	–
Pestalotiopsis hunanensis	CFCC 59540	*Pinus massoniana*	China	OR533579	–	OR539188	OR539193	–	–	–	–
Pestalotiopsis hydei	MFLUCC 20-0135 T	*Litsea petiolata*	Thailand	MW266063	–	MW251113	MW251112	–	–	–	–
Pestalotiopsis hydei	E-72-02	*Eucalyptus urograndis*	Brazil	KU926708	–	KU926712	KU926716	–	–	–	–
Pestalotiopsis hydei	E-72-03	*Eucalyptus urograndis*	Brazil	KU926709	–	KU926713	KU926717	–	–	–	–
Pestalotiopsis kandelicola	NCYUCC 19-0355 T	*Kandelia candel*	Taiwan, China	MT560722	–	MT563101	MT563099	–	–	–	–

Pestalotiopsis kandelicola	NCYUCC 19-0354	*Kandelia candel*	Taiwan, China	MT560723	–	MT563102	MT563100	–	–	–	–
Pestalotiopsis knightiae	CBS 114138 T	*Knightia* sp.	New Zealand	KM199310	–	KM199497	KM199408	–	–	–	–
Pestalotiopsis knightiae	CBS 111963	*Knightia* sp.	New Zealand	KM199311	–	KM199495	KM199406	–	–	–	–
Pestalotiopsis leucospermi	CBS 114489 T	*Leucospermum* cv.	America	MH553978	–	MH554396	MH554637	–	–	–	–
Pestalotiopsis linguae	ZHKUCC 22-0159 T	*Pyrrosia lingua*	China	OP094104	–	OP186110	OP186108	–	–	–	–
Pestalotiopsis linguae	ZHKUCC 22–0160	*Pyrrosia lingua*	China	OP094103	–	OP186109	OP186107	–	–	–	–
Pestalotiopsis lithocarpi	CFCC 55100 T	*Lithocarpus chiungchungensis*	China	OK339742	–	OK358503	OK358518	–	–	–	–
Pestalotiopsis lithocarpi	CFCC 55893	*Lithocarpus chiungchungensis*	China	OK339743	–	OK358504	OK358519	–	–	–	–
Pestalotiopsis lobata	CGMCC 3.23467 T	*Lithocarpus glaber*	China	OR247976	–	OR361451	OR381051	–	–	–	–
Pestalotiopsis lobata	LC15843	*Lithocarpus glaber*	China	OR247977	–	OR361452	OR381052	–	–	–	–
Pestalotiopsis loeiana	MFLUCC 22-0123 T	Unidentified host	Thailand	OP497988	–	OP737881	OP713769	–	–	–	–
Pestalotiopsis macadamiae	BRIP 63738b HT	*Macadamia integrifolia*	Australia	KX186588	–	KX186621	KX186680	–	–	–	–
Pestalotiopsis macadamiae	BRIP 63739a	*Macadamia integrifolia*	Australia	KX186589	–	KX186622	KX186681	–	–	–	–
Pestalotiopsis machili	CGMCC 3.23511 T	*Machilus* sp.	China	OR248003	–	OR361478	OR381078	–	–	–	–
Pestalotiopsis menhaiensis	CGMCC 3.18250 T	*Camellia sinensis*	China	KU252272	–	KU252401	KU252488	–	–	–	–
Pestalotiopsis menhaiensis	YN3A 2	*Camellia sinensis*	China	KX146685	–	KX146744	KX146803	–	–	–	–
Pestalotiopsis multicolor	CFCC 59981 T	*Taxus chinensis*	China	OQ626676	–	OQ714341	OQ714336	–	–	–	–
Pestalotiopsis multicolor	CGMCC 3.23171	*Camellia* sp.	China	KX894990	–	KX895207	KX895321	–	–	–	–
Pestalotiopsis nanningensis	CSUFTCC11 T	*Camellia oleifera*	China	OK493597	–	OK507967	OK562372	–	–	–	–
Pestalotiopsis nanningensis	CSUFTCC10	*Camellia oleifera*	China	OK493596	–	OK507966	OK562371	–	–	–	–
Pestalotiopsis oryzae	CBS 353.69 T	*Oryza sativa*	Denmark	KM199299	–	KM199496	KM199398	–	–	–	–
Pestalotiopsis oryzae	CBS 111522	*Telopea* sp.	America	KM199294	–	KM199493	KM199394	–	–	–	–
Pestalotiopsis parva	CBS 278.35 T	*Leucothoe fontanesiana*	Unknown	KM199313	–	KM199509	KM199405	–	–	–	–
Pestalotiopsis parva	CBS 265.37	*Delonix regia*	Unknown	KM199312	–	KM199508	KM199404	–	–	–	–
Pestalotiopsis photinicola	GZCC 16-0028 T	*Photinia serrulata*	China	OR230042	–	KY047662	OR381099	–	–	–	–
Pestalotiopsis photinicola	LC3637	*Camellia* sp.	China	KX894993	–	KX895210	KX895324	–	–	–	–

Pestalotiopsis photinicola	LC3640	*Camellia* sp.	China	KX894995	–	KX895212	KX895326	–	–	–	–
Pestalotiopsis phyllostachydis	ZHKUCC 23-0873 T	*Phyllostachys sulphurea*	China	OR343210	–	OR367675	OR367676	–	–	–	–
Pestalotiopsis phyllostachydis	CGMCC 3.23512 T	*Cratoxylum cochinchinense*	China	OR248005	–	OR361480	OR381080	–	–	–	–
Pestalotiopsis phyllostachydis	LC8772	*Cratoxylum cochinchinense*	China	OR248004	–	OR361479	OR381079	–	–	–	–
Pestalotiopsis pinicola	KUMCC 19-0183 T	*Pinus armandii*	China	MN412636	–	MN417509	MN417507	–	–	–	–
Pestalotiopsis pinicola	KUMCC19-0203	*Pinus armandii*	China	MN412637	–	MN417510	MN417508	–	–	–	–
Pestalotiopsis piraubensis	COAD 2165 T	*Psidium guajava*	Brazil	MH627381	–	MH643774	MH643773	–	–	–	–
Pestalotiopsis pruni	CGMCC 3.23507 T	*Prunus cerasoides*	China	OR248001	–	OR361476	OR381076	–	–	–	–
Pestalotiopsis pruni	LC15860	*Prunus cerasoides*	China	OR248002	–	OR361477	OR381077	–	–	–	–
Pestalotiopsis pruni	GUCC 23-0433	*Juglans regia*	China	PQ129398	–	PQ141033	PQ148983	–	–	–	–
Pestalotiopsis pruni	GUCC 23-0524	*Juglans regia*	China	PX425043	–	PX432021	PX432170	–	–	–	–
Pestalotiopsis rhizophorae	MFLUCC 17-0416 T	*Rhizophora apiculata*	Thailand	MK764283	–	MK764327	MK764349	–	–	–	–
Pestalotiopsis rhizophorae	MFLUCC 17-0417	*Rhizophora apiculata*	Thailand	MK764284	–	MK764328	MK764350	–	–	–	–
Pestalotiopsis rhodomyrti	HGUP 4230 T	*Rhodomyrtus tomentosa*	China	KF412648	–	KF412645	KF412642	–	–	–	–
Pestalotiopsis rhodomyrti	LC3413	*Camellia sinensis*	China	KX894981	–	KX895198	KX895313	–	–	–	–
Pestalotiopsis rosea	GUCC 195257	*Pinus* sp.	China	JX399005	–	JX399069	JX399036	–	–	–	–
Pestalotiopsis rosea	GUCC 23-0308	*Vicia faba*	China	PQ129350	–	PQ140985	PQ148935	–	–	–	–
Pestalotiopsis rosea	MFLUCC 12-0258 T	*Vicia faba*	China	PQ129351	–	PQ140986	PQ148936	–	–	–	–
Pestalotiopsis rosea	GUCC 23-0309	*Sabal mexicana*	China	ON180775	–	ON221533	ON221561	–	–	–	–
Pestalotiopsis sabal	ZHKUCC 22-0035 T	*Sabal mexicana*	China	ON180769	–	ON221527	ON221555	–	–	–	–
Pestalotiopsis sabal	ZHKUCC 22-0031	*Quercus variabilis*	China	ON007026	–	ON005043	ON005054	–	–	–	–
Pestalotiopsis shaanxiensis	CFCC 54958 T	*Quercus variabilis*	China	ON007027	–	ON005044	ON005055	–	–	–	–
Pestalotiopsis shaanxiensis	CFCC 57356	*Smilax china*	Thailand	OP497991	–	OP753376	OP762673	–	–	–	–
Pestalotiopsis smilacicola	MFLUCC 22-0125 T	*Smilax* sp.	Thailand	OP497989	–	OP737879	OP762674	–	–	–	–

Pestalotiopsis smilacicola	MFLUCC 22-0124	*Sonneratia apetala*	China	ON114184	-	ON086812	ON086816	-	-	-	-
Pestalotiopsis sonneratiae	CFCC 57394 T	*Sonneratia apetala*	China	ON114185	-	ON086813	ON086817	-	-	-	-
Pestalotiopsis sonneratiae	CFCC 57395	*Rhododendron delavayi*	China	OP082428	-	OP185514	OP185521	-	-	-	-
Pestalotiopsis telopeae	CBS 114161 T	*Telopea* sp.	Australia	KM199296	-	KM199500	KM199403	-	-	-	-
Pestalotiopsis telopeae	CBS 114137	*Protea* ‘Pink Ice’	Australia	KM199301	-	KM199559	KM199469	-	-	-	-
Pestalotiopsis terricola	CBS 141.69 T	Soil	Pacific Islands	MH554004	-	MH554438	MH554680	-	-	-	-
Pestalotiopsis thailandica	MFLUCC 17-1616 T	*Rhizophora apiculata*	Thailand	MK764285	-	MK764329	MK764351	-	-	-	-
Pestalotiopsis thailandica	MFLUCC 17-1617	*Rhizophora apiculata*	Thailand	MK764286	-	MK764330	MK764352	-	-	-	-
Pestalotiopsis trachycarpicola	GUCC 23-0190	*Trachycarpus fortunei*	China	JQ845947	-	JQ845946	JQ845945	-	-	-	-
Pestalotiopsis trachycarpicola	GUCC 23-0303	Unidentified host	China	JX399000		JX399064	JX399031	-	-	-	-
Pestalotiopsis trachycarpicola	CBS 442.67 T	*Chrysophyllum* sp.	China	JX399004	-	JX399068	JX399035	-	-	-	-
Pestalotiopsis trachycarpicola	CBS 911.96	*Rosa roxburghii*	China	PQ129324	-	PQ140959	PQ148909	-	-	-	-
Pestalotiopsis trachycarpicola	LC6633	*Vigna unguiculata*	China	PQ129349	-	PQ140984	PQ148934	-	-	-	-
Pestalotiopsis sp.	GUCC 23-0365	*Coffea* sp.	Kenya	KM199302	-	KM199502	KM199395	-	-	-	-
Pestalotiopsis sp.	GUCC 23-0367	Raw material from agar-agar	Unknown	KM199303	-	KM199503	KM199396	-	-	-	-
Pestalotiopsis ampelopsidis	**YzU 251456 T**	***Nekemias grossedentata***	**China**	**PZ343082**	-	**PZ388308**	**PZ388310**	-	-	-	-
Pestalotiopsis ampelopsidis	**YzU 251461**	***Nekemias grossedentata***	**China**	**PZ343083**	-	**PZ388309**	**PZ388311**	-	-	-	-
Pestalotiopsis dilucida	LF444 T	*Camellia sinensis*	China	KX894961	-	KX895178	KX895293	-	-	-	-

Notes: New species established in this study are shown in bold. Those marked “T” in the table are represented as ex-type or ex-epitype strains, and those marked “NT” are represented as ex-neotype cultures; “HT” are represented as ex-holotype
